# Maternal opioid use and hepatitis C infection disrupt the placental immune landscape and structure

**DOI:** 10.1172/jci.insight.199606

**Published:** 2026-03-17

**Authors:** Heather E. True, Brianna M. Doratt, Sheridan B. Wagner, Delphine C. Malherbe, Nathan R. Shelman, Mahdi Eskandarian Boroujeni, Cynthia Cockerham, John M. O’Brien, Ilhem Messaoudi

**Affiliations:** 1 Department of Microbiology, Immunology, and Molecular Genetics, College of Medicine,; 2 Department of Pathology, College of Medicine, and; 3 Department of Obstetrics & Gynecology, College of Medicine, University of Kentucky, Lexington, Kentucky, USA.

**Keywords:** Clinical Research, Immunology, Reproductive biology, Cellular immune response, Obstetrics/gynecology, Transcriptomics

## Abstract

Maternal opioid use disorder (OUD) poses substantial risks to maternal and fetal health. These adverse outcomes are believed to be mediated, in part, by changes in placental structure and function; however, few studies have addressed this question. Here, we utilized flow cytometry, histology, and spatial and single-cell transcriptomics to uncover the impact of OUD on placental tissues. Given that half of individuals with chronic OUD contract hepatitis C (HCV), we further stratified our findings by maternal HCV status. Our results indicate that OUD leads to higher incidence of vascular malperfusion accompanied by increased levels of inflammatory markers and dysregulated secretion of placental development factors. Spatial transcriptomics revealed that OUD disrupts the communication between trophoblasts and immune cells important for placental vascular development. Additionally, CellChat analysis revealed aberrant vascular remodeling, neuropeptide, and chemotactic signaling across trophoblast, endothelial, and myeloid cells. Processes associated with tissue homeostasis and repair were also upregulated across trophoblasts and leukocytes. In addition, placental leukocytes were rewired toward regulatory/tissue surveillant phenotypes. Finally, frequencies and responses to ex vivo stimulation of decidual macrophages and cytolytic NK cells, critical for tissue remodeling and fetal tolerance, were decreased. Altogether, these results highlight substantial disruptions to placental health by maternal OUD.

## Abstract 2

Opioid use disorder during pregnancy disrupts placenta health with implications for adverse impacts to maternal and fetal health.

## Introduction

Opioid use disorder (OUD) continues to be a substantial public health concern, with national overdose deaths nearly quadrupling from 2012 to 2022 (1). Drug overdose mortality rates in pregnancy nearly doubled from 2018 to 2021 (2), and OUD in pregnancy has been linked to adverse outcomes for both pregnant women and fetuses. Specifically, OUD has been associated with higher rates of maternal mortality, placental abruption, preterm labor, oligohydramnios, increased risk of fetal demise, poor fetal growth, preterm birth, birth defects, and impaired neurodevelopment (3–8).

These adverse outcomes are believed to be mediated by changes in placental vascular development and function (9, 10). The placenta is composed of maternal- and fetal-derived components, termed the decidua basalis and chorionic villous, respectively (11). The decidua basalis gradually thins throughout gestation but maintains an essential role by providing a scaffolding for the growing chorionic villous and fetus while producing factors to initiate contractions (12, 13). The chorionic villous is the main site for angiogenesis, where dense networks of villi with newly formed capillaries increase the surface area available for gas and nutrient exchange between the chorion and the maternal uterine lining (14). Therefore, the placenta is a key mediator of fetal/maternal interaction by providing signals that regulate fetal growth and tolerance (15).

A healthy placenta is composed of trophoblast, endothelial, and stromal cells that facilitate fetal development, placental circulation, and hormone/cytokine signaling (13). Trophoblast cells are specialized fetal epithelial cells that differentiate from the outer layer of the developing embryo, maintain placental integrity, and mediate the complex interplay with the maternal immune system (11). Placental endothelial cells maintain the integrity of the placental barrier by signaling to trophoblasts, stromal cells, and immune cells to promote vascular remodeling, optimize maternal blood flow, and mount antiviral defenses (16, 17). Placental stromal cells have potent immunosuppressive properties that prevent immune-mediated rejection of the fetus (15, 18). In addition, the decidua is composed of maternally derived immune cells, including T cells, NK cells, macrophages, and DCs (19). Decidual immune cells play critical roles in maintaining immune tolerance (20), promoting fetal growth (21), and responding to infections at the maternal-fetal interface (22, 23).

Immune cells of the chorionic villous consist exclusively of primitive fetal yolk sac–derived macrophages called Hofbauer cells (HBCs) and infiltrating placenta-associated maternal monocytes and macrophages (PAMMs). HBCs are critical for placental morphogenesis, maintenance of fetal tolerance, and protection of the fetus from infection and inflammation (24, 25). PAMMs are a heterogeneous population of maternal monocytes and macrophages. PAMM1A cells are the most abundant, are morphologically similar to macrophages, adhere to sites of placental injury, secrete tissue repair factors, and regulate trophoblast function (26). PAMM1B cells are monocytes with unique gene signatures from circulating blood monocytes and are thought to be PAMM1A precursors (26). Finally, PAMM2 macrophages are present in low abundance in healthy pregnancies and are considered contaminating macrophages from the maternal decidua (26).

Few studies have addressed the impact of opioids on placental structure and function (27, 28). Pregnant individuals treated with opioid maintenance therapy are at a markedly higher risk for placental dysmaturity, characterized by irregular villi, decreased syncytial knots, hypervascularity, and villous edema (28, 29). Additionally, pregnant individuals who receive prescription opioids during pregnancy (regardless of gestational age) are at a higher risk of placental insufficiency and/or ischemia (30). However, the mechanisms by which opioids result in these adverse outcomes remain poorly understood. Furthermore, pregnancies exposed to opioids are often complicated by maternal hepatitis C (HCV) infection, as over half of people who inject drugs develop HCV within 5 years (31). Additional studies are necessary to uncover the impact of maternal OUD with and without HCV infection (OUD±HCV) on placental structure and immunity.

Thus, we collected maternal (decidua basalis) and fetal (chorionic villous) placental tissues from healthy pregnant individuals and individuals with severe OUD±HCV, and then subjected the tissues to a systems biology approach to uncover the impact of OUD±HCV on the structure, function, and immune landscape of the maternal-fetal interface. H&E-stained placental tissues were reviewed for histological pathologies and further interrogated by Visium spatial transcriptomics. Finally, given the high prevalence of HCV infection in this cohort, we stratified our findings by maternal HCV status.

## Results

### Maternal opioid use and HCV infection were associated with adverse maternal-fetal outcomes.

**Figure 1:**
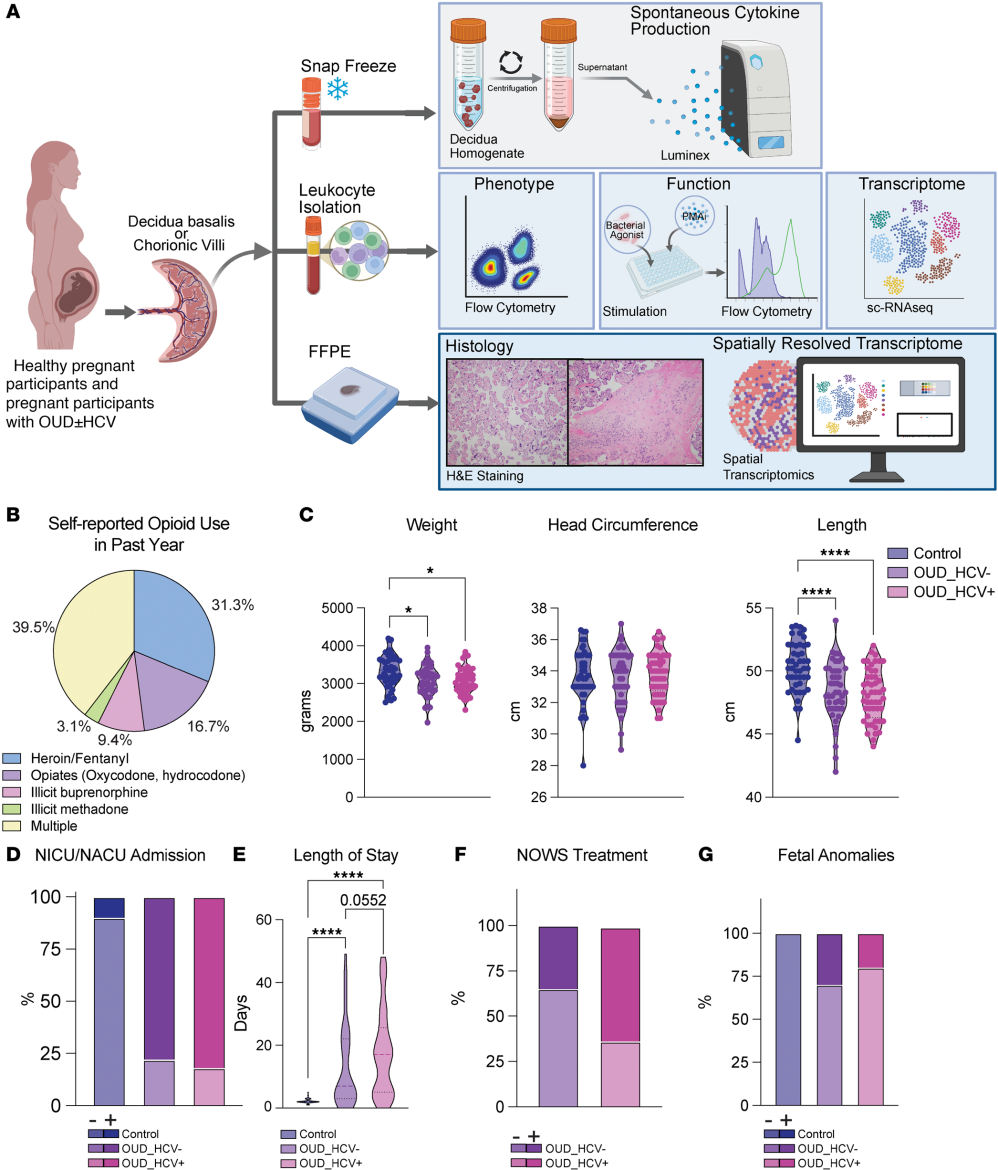
Maternal OUD±HCV leads to adverse maternal-fetal outcomes. (**A**) Experimental design. (**B**) Pie chart depicting self-reported substance use at enrollment during the previous 1 year. “Multiple” indicates the use of 2 or more of the listed opioid substances. (**C**) Violin plots of the indicated newborn measurement. One-way ANOVA with Tukey’s HSD test. (**D**) Stacked bar plot depicting the frequency of NICU/neonatal abstinence care unit (NACU) admission in newborns; χ^2^ test. (**E**) Violin plot of NICU/NACU length of stay. One-way ANOVA with Tukey’s HSD test. (**F** and **G**) Stacked bar plots showing the frequency of (**F**) newborns who received NOWS treatment and (**G**) newborns presenting with fetal anomalies; χ^2^ test. Control *n* = 48; OUD_HCV^–^ *n* = 50; OUD_HCV^+^ *n* = 46. *P < 0.05; ****P < 0.0001.

Decidua and chorionic villous tissues were isolated from placentas of full-term pregnancies (>37 weeks) of participants with and without OUD (Figure 1A). All participants in the OUD group were screened per the Diagnostic and Statistical Manual of Mental Disorders, fifth edition (DSM-V) criteria for severe OUD, as defined by meeting 4 or more criteria at enrollment (Table 1). All participants in the OUD group reported a history of illicit opioid use (heroin, fentanyl, and/or nonprescribed opiates, buprenorphine, and methadone products), with 39.5% of participants using more than one opioid (Figure 1B). Additionally, participants with OUD were stabilized on medication-assisted treatments (opioid agonist therapies; buprenorphine or methadone) at the time of delivery. We then stratified participants based on their HCV status at the time of delivery, determined by HCV IgG levels and viral loads (Table 1). Of the total 96 participants with OUD screened for HCV, 50 participants were positive for anti-HCV IgG, indicating history of infection, and 21 had a viral load detected by RT-PCR, indicating active HCV infection at delivery (Table 1). Polysubstance use was noted in the OUD groups, with rates of alcohol and illicit benzodiazepines highest in the group without HCV infection (OUD_HCV^–^) (Table 1). Maternal age, prepregnancy BMI, gestational age, and mode of delivery were comparable (Table 1). Additionally, induction of labor was more frequent in both OUD groups, whereas gestational hypertension and preeclampsia were not different by group (Table 1). The control group also had more non-White participants than both OUD groups (Table 1).

Maternal OUD exerts acute and long-term adverse outcomes for newborns, including growth restriction, developmental delays, and increased likelihood of chronic diseases (4). In line with these observations, OUD was associated with smaller weight and length in newborns (Figure 1C and Table 1), who were more likely to be admitted to a neonatal intensive care unit (NICU) (Figure 1D) for a protracted length of time (Figure 1E). These findings were exacerbated by maternal HCV infection (Figure 1, D and E), as was the need for pharmacological treatment (morphine) for neonatal opioid withdrawal syndrome (NOWS) (Figure 1F and Table 1). Finally, fetal anomalies (birth defects) were more abundant in newborns of participants with OUD (Figure 1G and Table 1). These findings highlight a substantial impact of maternal OUD on fetal outcomes that is further exacerbated by HCV infection.

### Altered inflammatory milieu and immune profiles of the decidua basalis with maternal OUD±HCV.

**Figure 2:**
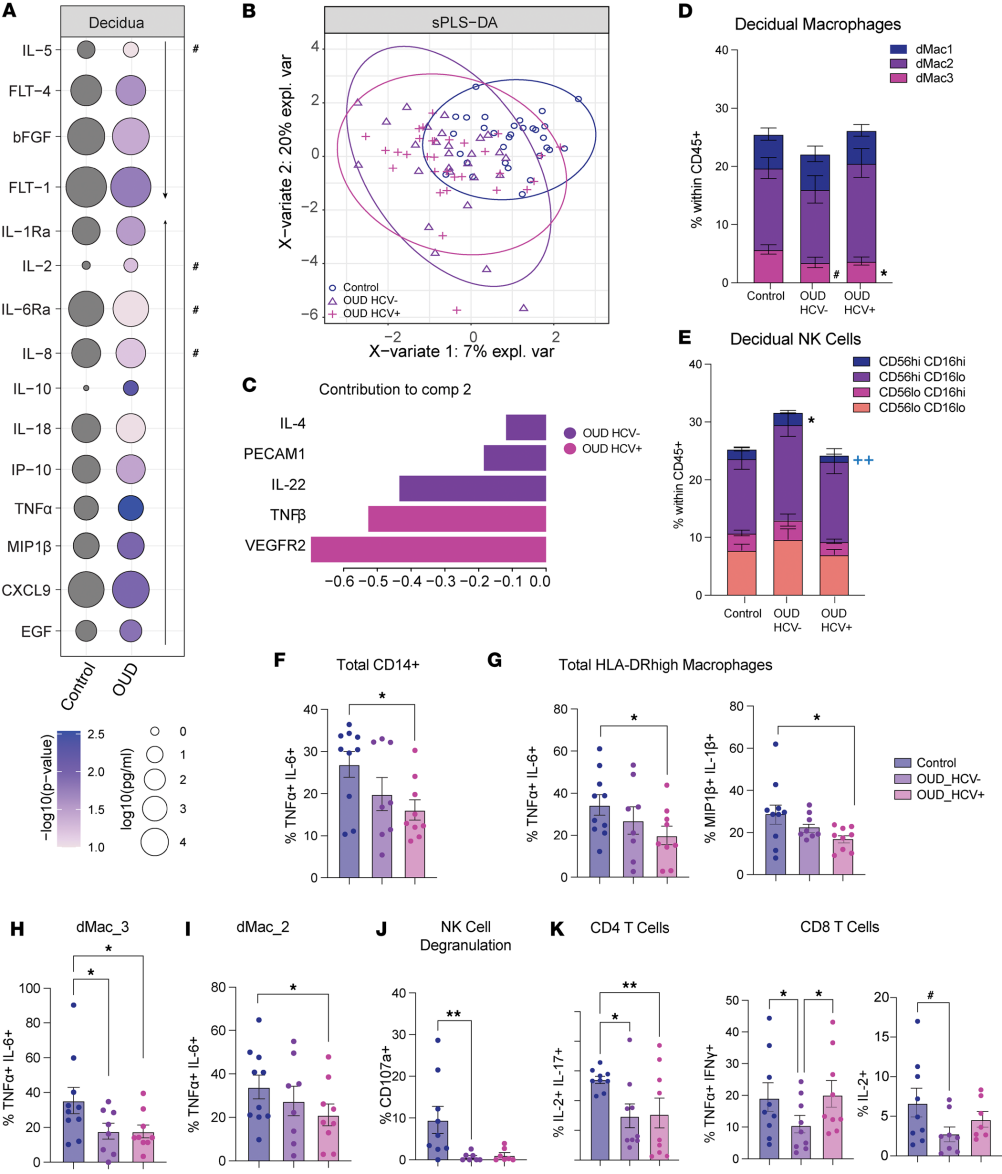
Decidual immune cell phenotype and function are altered by maternal OUD±HCV. (**A**) Bubble plot of protein concentration in decidua tissue homogenate supernatant. Size of the bubble represents log_10_ (pg/mL) and color represents *P* value. Arrows indicate direction of change in concentrations (increase or decrease) compared with controls. Unpaired Welch’s *t* tests (2-tailed) for each analyte. (**B**) sPLSDA plot of protein concentration in decidua tissue homogenate. (**C**) Bar plot of markers delineating groups along sPLSDA component 2. (**D** and **E**) Stacked bar plots of subset frequencies for (**D**) HLA-DR^hi^ decidual macrophages and (**E**) NK cells. Each subset was assessed using a 1-way ANOVA with Tukey’s HSD test. (**F**–**K**) Responses to stimulations displayed after subtraction of the baseline, assessed using 1-way ANOVA with Tukey’s HSD test. (**F**–**I**) Bar plots of percentage of cells responding to bacterial TLR ligand stimulation from (**F**) total CD14^+^ cells, (**G**) total HLA-DR^hi^ macrophages, (**H**) dMac_3, and (**I**) dMac_2 cells. (**J**) Bar plot of the percentage of NK cells expressing CD107a in response to PMAi stimulation. (**K**) Bar plot of percentage response of CD4^+^ (left) and CD8^+^ (right) T cells to PMAi stimulation. Symbols in black denote comparisons to control; blue symbols denote significance between OUD_HCV^–^ and OUD_HCV^+^ groups. For Luminex data: control *n* = 30; OUD_HCV^–^ *n* = 26; OUD_HCV^+^ *n* = 31. For flow cytometry data: control *n* = 15; OUD_HCV^–^ *n* = 14; OUD_HCV^+^ *n* = 10. ^#^*P* < 0.1, *P < 0.05, ^**/++^*P* < 0.01.

The placenta undergoes dynamic changes throughout gestation to support fetal growth while maintaining fetal tolerance (32, 33). These changes are tightly regulated by immune mediators and growth factors (33). Measurements of immune mediators and angiogenesis factors in decidua tissue homogenate revealed a significant increase in proinflammatory cytokines (TNF-α, IL-6RA, IL-1Ra, IL-18, IL-2), chemokines (IP-10, MIP-1β, CXCL9, IL-8), and EGF with OUD (Figure 2A). However, levels of TH2 cytokine IL-5 and angiogenesis factors (FLT-1/4) were reduced compared with the control group (Figure 2A). We then carried out a sparse partial least squares differential analysis (sPLSDA) to identify factors that separate the 3 groups (Figure 2, B and C). Factors including IL-4, PECAM1, and IL-22 delineated the OUD_HCV^–^ group; those that differentiated the OUD_HCV^+^ group included TNF-β and VEGFR2 (Figure 2C). These data indicate that maternal OUD is associated with increased decidual inflammation that is amplified by HCV coinfection.

It is well established that biomarkers of placental dysfunction can be detected in maternal circulation (34). Therefore, we measured levels of placental development markers in maternal plasma. We detected lower levels of PDGF-AA, FGF1, angiopoetin-1, ANGPTL-4/6, and FLT-1 with maternal OUD (Supplemental Figure 1A; supplemental material available online with this article; https://doi.org/10.1172/jci.insight.199606DS1). Decreased levels of these markers in maternal plasma generally reflect impaired placental vascular development and reduced fetal nutrient and oxygen supply (35). Furthermore, we detected elevated levels of PAPP-A, PECAM-1, FLT-4, MMP-8, and GM-CSF in maternal plasma in the OUD group versus the control group (Supplemental Figure 1A). High levels of these markers have been associated with placental dysfunction and hypertensive disorders in pregnancy (36) and are in line with increased rates of gestational hypertension in both OUD cohorts (Table 1). Further sPLSDA delineating the OUD_HCV^+^ group included tenascin C, EGF, FGF-1, IL-6RA, and PDGF-AA; those that differentiated the OUD_HCV^–^ group included c-kit, angiopoietin-1, FLK, and MMP8 (Supplemental Figure 1C). Finally, PECAM1, MMP9, HGF, FLT4, ANGPTL3/4/6, and FLT1 levels segregated the control from the OUD groups (Supplemental Figure 1C). Altogether, these observations highlight that maternal OUD leads to a proinflammatory milieu and elevated placental repair signaling.

We next assessed the decidual immune cell compartment using flow cytometry (Supplemental Figure 1D). Although no significant differences in the abundance of total monocytes, DCs, NK cells, or T cells were observed (Supplemental Figure 1E), there were significant shifts in the frequency of HLA-DR^hi^ decidual macrophage and NK cell subsets with maternal OUD±HCV (Figure 2, D and E). Specifically, the frequency of dMac_3 macrophages (FOLR2^hi^S100A8/9^lo^CD9^–^), which are mature decidual macrophages important for tissue remodeling and clearance of cell debris in late pregnancy (19), was reduced with maternal OUD (Figure 2D). Furthermore, we report a decrease in cytolytic CD56^hi^CD16^hi^ NK cells, recently described as critical for regulating immune responses and supporting placentation in healthy pregnancies (37), in OUD_HCV^+^ participants (Figure 2E).

To evaluate differences in the functional capacity, we measured responses to ex vivo stimulation (Figure 1A). Decreased responses to bacterial ligands by total decidual macrophages was noted with maternal OUD and HCV infection (Figure 2F), driven by dampened responses generated by HLA-DR^hi^ macrophages (Figure 2G), namely dMac_3 and dMac_2 subsets (Figure 2, H and I). Additionally, degranulation by decidual (dNK) cells in response to stimulation by PMA/ionomycin (PMAi) was decreased with maternal OUD (Figure 2J). Finally, although no differences were observed in the abundance of decidual CD4^+^ or CD8^+^ T cells (Supplemental Figure 1E), T cell responses to polyclonal stimulation by PMAi were also decreased with maternal OUD (Figure 2K). Altogether, these findings indicate that maternal OUD±HCV alters the abundance and function of immune cells in the decidua.

### Altered inflammatory milieu and immune profiles of chorionic villous (fetal) placental tissues with maternal OUD±HCV.

Next, we compared differences in inflammatory and angiogenesis factors in villous tissue homogenate (Supplemental Figure 3A). Levels of IL-12p70 and IL-7 were increased, and those of IL-6 and IL-8 were decreased with maternal OUD (Supplemental Figure 3A). Changes in levels of these cytokines have been associated with preeclampsia and preterm birth (38, 39). We next carried out an sPLSDA to extract factors that distinguish between all groups (Supplemental Figure 3, B and C). Notable markers that delineated the OUD_HCV^+^ group included TNF-β, IFN-γ, GM-CSF, IFNα-2, and eotaxin, whereas IL-17A, IL-12p70, IL-7, IL-13, and IL-4 delineated the OUD_HCV^–^ group (Supplemental Figure 1B). However, IL-10, TNF-α, EGF, and IL-1α levels segregated the control group from the OUD groups (Supplemental Figure 1B). These findings highlight that maternal OUD±HCV shifts the placental milieu toward a hyperinflammatory state.

We then used flow cytometry to characterize the immune landscape of the chorionic villous (Figure 1A and Supplemental Figure 3D). No differences were found in the abundance of HBCs with maternal OUD regardless of HCV status (Supplemental Figure 3E). The frequency of PAMM1A cells increased in the OUD_HCV^+^ group compared with controls and the OUD_HCV^–^ group, whereas that of PAMM2 cells decreased (Supplemental Figure 3E). A modest decline in PAMM1B cell abundance in the OUD_HCV^+^ relative to the OUD_HCV^–^ group was seen (Supplemental Figure 3E). No differences were observed in the phagocytosis potential (Supplemental Figure 3F and Supplemental Figure 2D) or cytokine response of chorionic villous macrophages after stimulation with bacterial TLR ligands (Supplemental Figure 3G). These data suggest that maternal OUD and HCV infection have a more profound impact on the inflammatory milieu and immune cell landscape of the decidua relative to villous tissues.

### Maternal OUD rewires the transcriptional profiles of placental macrophages toward immune tolerant and tissue repair phenotypes.

**Figure 3:**
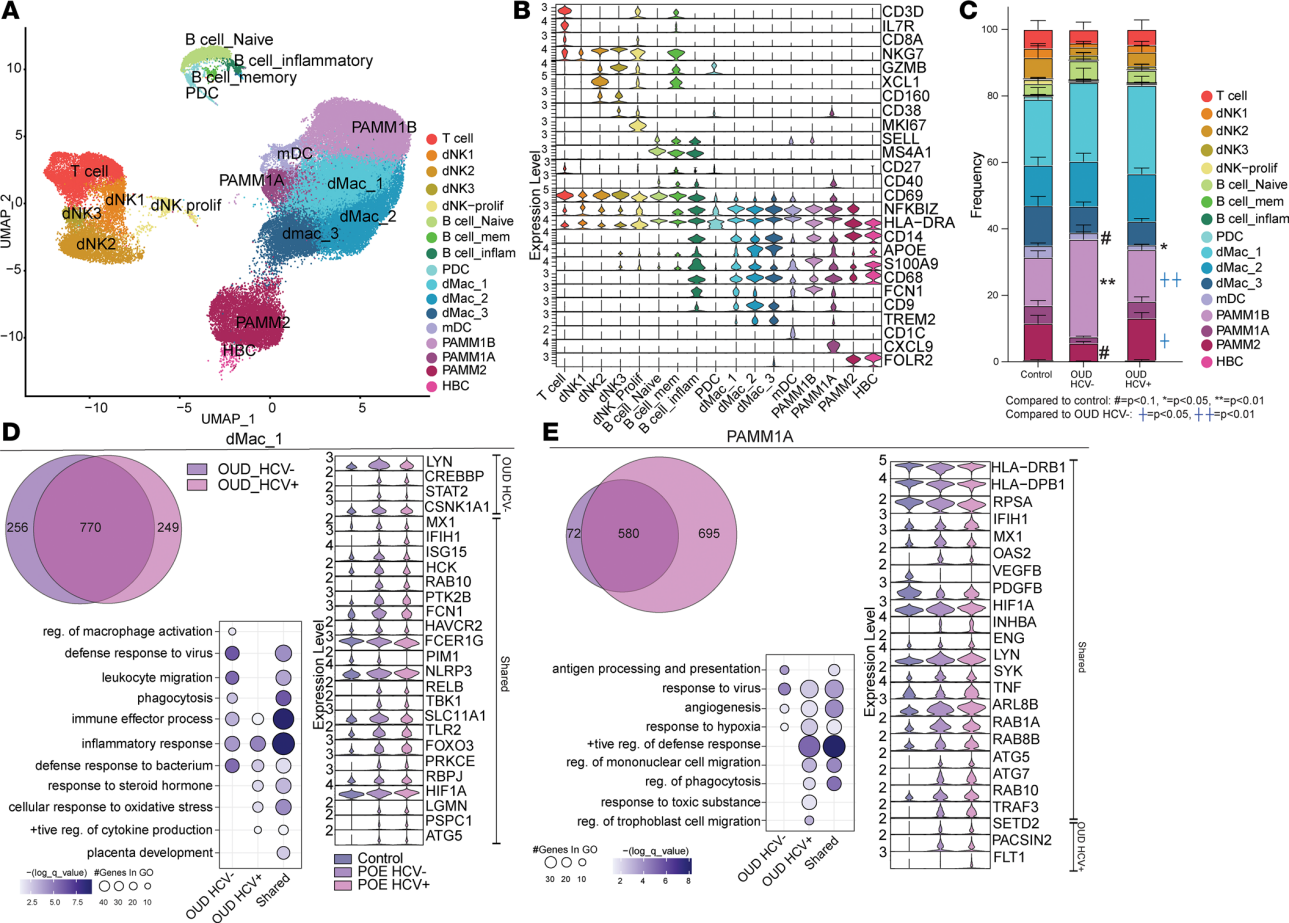
Maternal OUD±HCV rewires the transcriptional profiles of placental macrophages. (**A**) UMAP of placental immune cell (73,256 cells). (**B**) Violin plots of cluster marker genes. (**C**) Stacked bar graph of cluster frequencies. Control 15,069 cells; OUD_HCV^–^ 26,381 cells; OUD_HCV^+^ 31,806 cells. Each cluster was assessed using 1-way ANOVA with Tukey’s HSD test. (**D** and **E**) Left: Venn diagram of DEG overlap from OUD_HCV^–^ versus control (purple) and OUD_HCV^+^ versus control (pink) comparisons. Middle: bubble plot of select GO terms from DEG enrichments using Metascape default settings. Size represents gene number and color represents log_10_ (*q* value). Right: violin plots of representative DEGs for (**D**) dMac_1 and (**E**) PAMM1A clusters. For scRNA-Seq, *n* = 3/group. ^#^*P* < 0.1, *P < 0.05, **P <0.01, ^**/++^*P* < 0.01.

Using single-cell RNA-Seq (scRNA-Seq), we profiled the transcriptome of CD45^+^ FACS-isolated placental leukocytes from decidua and chorionic villous tissues and integrated these datasets to construct a comprehensive transcriptional profile of the total placental immune landscape (Figure 1A). We identified 17 immune cell clusters encompassing previously defined populations from term decidua (19) and chorionic villous (40) (Figure 3, A and B, and Supplemental Table 1). Specifically, we identified T cells (*CD3^+^IL7R^+^CD8A^+^*); 4 populations of dNK cells (dNK1: *NKG7^+^*, dNK2: *NKG7^+^GZMB^hi^XCL1^+^CD160^+^*, dNK3: *NKG7^+^GZMB^lo^CD160^+^*, and dNK proliferating: *NKG7^+^MK67^+^*); and 3 B cell populations (B cell naive: *MS4A1^+^*, B cell memory: *MS4A1^+^ CD27^+^*, and B cell inflammatory: *MS4A1^+^CD69^+^HLA-DRA^+^*) (Figure 3, A and B). Among innate immune cells, we delineated 2 DC populations, plasmacytoid and myeloid (pDC: *CD38^+^GZMB^+^*, mDC: *CD1C^+^CD38^+^CD68^+^*); 3 *CD14^+^HLA-DR^hi^* decidual macrophage populations (dMac_1: *FCN1^+^*, dMac_2: *CD9^+^TREM2^+^*, dMac_3: *FOLR2^+^TREM2^+^*), as well as PAMM (PAMM1B: *HLA-DRA^+^S100A9^+^FCN1^+^SELL^+^*, PAMM1A: *HLA-DRA^+^CD9^+^CXCL9^+^*, PAMM2: *HLA-DRA^+^FOLR2^+^*) and HBC (*HLA-DRA^lo^FOLR2^+^NFKBIZ^–^*) subsets (Figure 3, A and B). We saw a marginal decrease in mDC and PAMM2 cells in the OUD_HCV^–^ compared with the control group, whereas frequencies of PAMM1B cells were expanded (Figure 3C). These shifts were modulated by maternal HCV infection (Figure 3C).

We next identified differentially expressed genes (DEGs) and performed functional enrichment to uncover the impact of maternal OUD±HCV on the transcriptional profiles of placental immune cells. Given the shifts in placental macrophage frequency, phenotype, and functional responses with maternal OUD±HCV, we focused our DEG analysis on the 6 macrophage clusters, which also demonstrated the highest number of DEGs. Large overlaps in DEGs were observed between both OUD groups within dMac clusters. Within dMac_1 and dMac_2, genes mapping to processes critical for host defense, inflammation, and stress responses were upregulated compared with controls (Figure 3D and Supplemental Figure 4A). Genes involved in antimicrobial defense (*MX1*, *IFIH1*, *ISG15*, *TLR2*), oxidative stress response (*FOXO3*, *RBPJ*, *HIF1A*, *PPARD*, *SRC*), and cytokine signaling (*RELB*, *IL6R*, *TGFBR1/2*, *MAPK1/8*) were downregulated in the OUD groups compared with the control group (Figure 3D and Supplemental Figure 4A). Genes linked to leukocyte migration (*RAB10*, *PTK2B*, *VAV3*, *ITGAV*) and phagocytosis (*HAVCR2*, *FCN1*) were upregulated in both dMac_1 and dMac_2 clusters (Figure 3D and Supplemental Figure 4A), while genes linked to placental development (*LGMN*, *PSPC1*, *ATG5*) were upregulated in the dMac_1 cluster only (Figure 3D). However, genes associated with immune effector functions (*FCER1G*, *CD81*, *TNF*, *PDGFB*) were downregulated in both OUD groups (Figure 3D and Supplemental Figure 4A). Notably, genes important for regulating macrophage activation (*CREBBP*, *STAT2*, *CSNK1A1*) were only upregulated in the OUD_HCV^–^ group, indicating that OUD alone uniquely perturbs key activation checkpoints. OUD±HCV reprograms dMac_1 and dMac_2 subsets toward an IFN-stimulated, pathogen-responsive state (Figure 3D and Supplemental Figure 4A).

Within the dMac_3 cluster, genes related to tissue remodeling and development (*MAP3K5*, *PIK3AP1*, *RAF1*), apoptosis and endocytosis (*TRAF3*, *RIPK2*, *DAPK1*, *AP3B1*), host defense (*IFIH1*, *IFIT3*, *IFI6*, *TLR2*, *TLR1*, *NLRP3*), and inflammatory signaling (*TNFAIP3*, *IL6R*, *SYK*, *IFNGR2*, *FNIP2*, *PPARD*, *FOXO3*) were upregulated with OUD regardless of HCV status (Supplemental Figure 4B). Upregulated DEGs unique to the OUD_HCV^–^ group mapped to cytokine signaling and production (*IRAK2*, *STAT1*, *JAK2*) (Supplemental Figure 4B). However, DEGs unique to the OUD_HCV^+^ group were enriched for regulation of acute inflammatory responses (*TBK1*, *IRAK3*, *FHIT*) (Supplemental Figure 4B). These transcriptional signatures were consistent with enhanced placental remodeling and heightened inflammatory signaling in OUD-exposed pregnancies, but HCV infection skewed dMac_3 toward tolerance.

There was a strong overlap in shared DEGs by both OUD groups in the PAMM1B cluster (Supplemental Figure 3C), whereas most of the DEGs identified in the PAMM1A and PAMM2 clusters were unique to OUD_HCV^+^ (Figure 3E and Supplemental Figure 4D). In the PAMM1A subset, DEGs upregulated in both OUD groups mapped to response to hypoxia (*HIF1A*, *INHBA*, *ENG*), antiviral defense (*IFIH1*, *MX1*, *OAS2*, *TRIM22*), regulation of defense responses (*LYN*, *SYK*, *TNF*), cell migration (*ARL8B*, *RAB1A/8B*), and phagocytosis (*ATG5/7*, *RAB10*) (Figure 3E). Downregulated genes enriched to antigen processing and presentation (*HLA-DRB1*, *HLA-DPB1*, *RPSA*) and angiogenesis (*VEGFB*, *PDGFB*) (Figure 3E). DEGs upregulated in the PAMM1A cluster with HCV coexposure mapped to response to toxic substances (*SETD2*, *PACSIN2*) and regulation of trophoblast cell migration (*FLT1*) (Figure 3E). These findings indicate that, despite blunted responses to ex vivo stimulation, PAMM1A cells were transcriptionally primed for defense responses, consistent with an immune tolerant phenotype (Figure 2, F and G). Moreover, HCV coinfection skewed PAMM1A cells toward enhanced responses to toxic stimuli and dysregulation of trophoblast migration.

Analysis of the PAMM1B cluster showed that DEGs shared across both OUD groups were important for tissue repair and placental development (*THBS1*, *MYH9*, *ADAM17*, *PECAM1*), differentiation and migration (*BCL6*, *NOTCH2*, *DOCK2*, *ITGB1*, *ITGAV*), regulation of inflammation (*NFKB1*, *PTGS2*, *REL*), as well as responses to hormones (*EP300*, *FOXP1*, *SP3*) and viruses (*IFI16*, *STAT3*, *IL6ST*) (Supplemental Figure 4C). With OUD alone, the PAMM1B cluster demonstrated altered expression of genes linked to macrophage activation (*AOAH* and *LYN* upregulated; *ANXA1* and *GSTP1* downregulated). HCV coinfection was associated with reduced expression of genes involved in cellular responses to toxic substances (*S100A8/12*, *GNAS*) (Supplemental Figure 4C). Overall, OUD was associated with rewiring of PAMM1B toward enhanced tissue repair, impaired macrophage activation, and suppressed antiviral responses.

Finally, DEGs upregulated with OUD in the PAMM2 subset mapped to responses to xenobiotic stimuli (*NFE2L2*, *ABCA1*, *TOP1*), whereas genes associated with tissue homeostasis, immune effector functions, and cell signaling were downregulated (Supplemental Figure 4D). Notably, genes linked to regeneration (*GSN*, *PYCARD*, *SPP1*), phagocytosis and exocytosis (*CTSB*, *FCER1G*, *CD74*, *SYNGR2*, *CORO1A*), regulation of Wnt signaling (*JUN*, *JUNB*, *CYBA*), immune effector process (*FCGR3A*, *SPI1*, *ITGB2*), and macrophage activation (*CD81*, *C5AR1*) were all decreased (Supplemental Figure 4D). Several upregulated DEGs unique to the HCV-exposed group within the PAMM2 cluster mapped to regulation of smooth muscle cell proliferation (*BMPR2*, *KMT2E*, *PPP3CA*), placental development and barrier function (*RUNX1*, *ARL8B*, *DOCK4*, *VAV3*), TLR signaling (*TLR1*, *NFKB1*, *FOXP1*), and responses to ischemia (*RAP1A/B*, *LYN*, *USP47*) and morphine (*SYK*, *PRDM1*) (Supplemental Figure 4D). Together, these data suggest that barrier integrity and tissue homeostasis functions of PAMM2 may be compromised with OUD and HCV exposure.

### Vascular malperfusion and inflammation are abundant in placenta with maternal OUD±HCV exposure.

**Figure 4:**
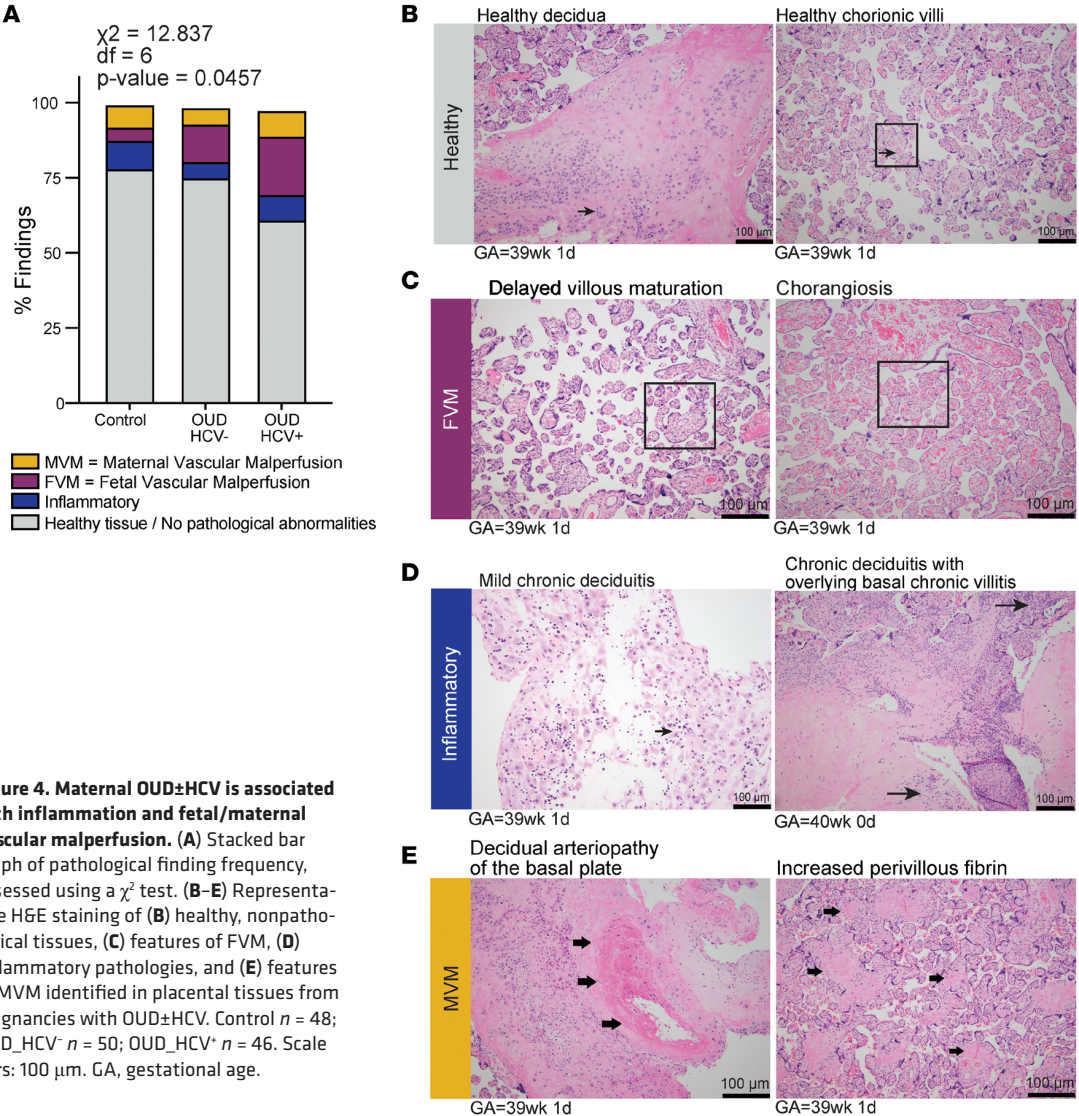
Maternal OUD±HCV is associated with inflammation and fetal/maternal vascular malperfusion. (**A**) Stacked bar graph of pathological finding frequency, assessed using a χ^2^ test. (**B**–**E**) Representative H&E staining of (**B**) healthy, nonpathological tissues, (**C**) features of FVM, (**D**) inflammatory pathologies, and (**E**) features of MVM identified in placental tissues from pregnancies with OUD±HCV. Control *n* = 48; OUD_HCV^–^ *n* = 50; OUD_HCV^+^ *n* = 46. Scale bars: 100 μm. GA, gestational age.

Disruptions in placental structure have been shown to directly affect placental immunity (41). Therefore, we reviewed histopathological findings of decidua and surrounding chorionic villous tissues from control and OUD±HCV groups (Figure 1A). Healthy, noninflamed maternal decidua was evident in control samples (Figure 4, A and B, arrows) and adjacent chorionic villous tissues exhibiting appropriate maturation and vascularity for gestation (Figure 4B, box and arrows). An increase in pathological findings was associated with OUD±HCV (Figure 4A), which was driven by fetal vascular malperfusion (FVM), including accelerated villous maturation and chorangiosis (Figure 4C). Other notable findings included features of deciduitis/villitis (Figure 4D, arrows) and maternal vascular malperfusion (MVM), including decidual arteriopathy of the basal plate and sites of increased perivillous fibrin (Figure 4E, arrows). Overall, these findings suggest a detrimental impact on placental development with OUD±HCV, notably placental hypoperfusion and hypoxia in the OUD_HCV^+^ group.

### Disruption of the spatially resolved transcriptional landscape of placental cells with maternal OUD.

**Figure 5:**
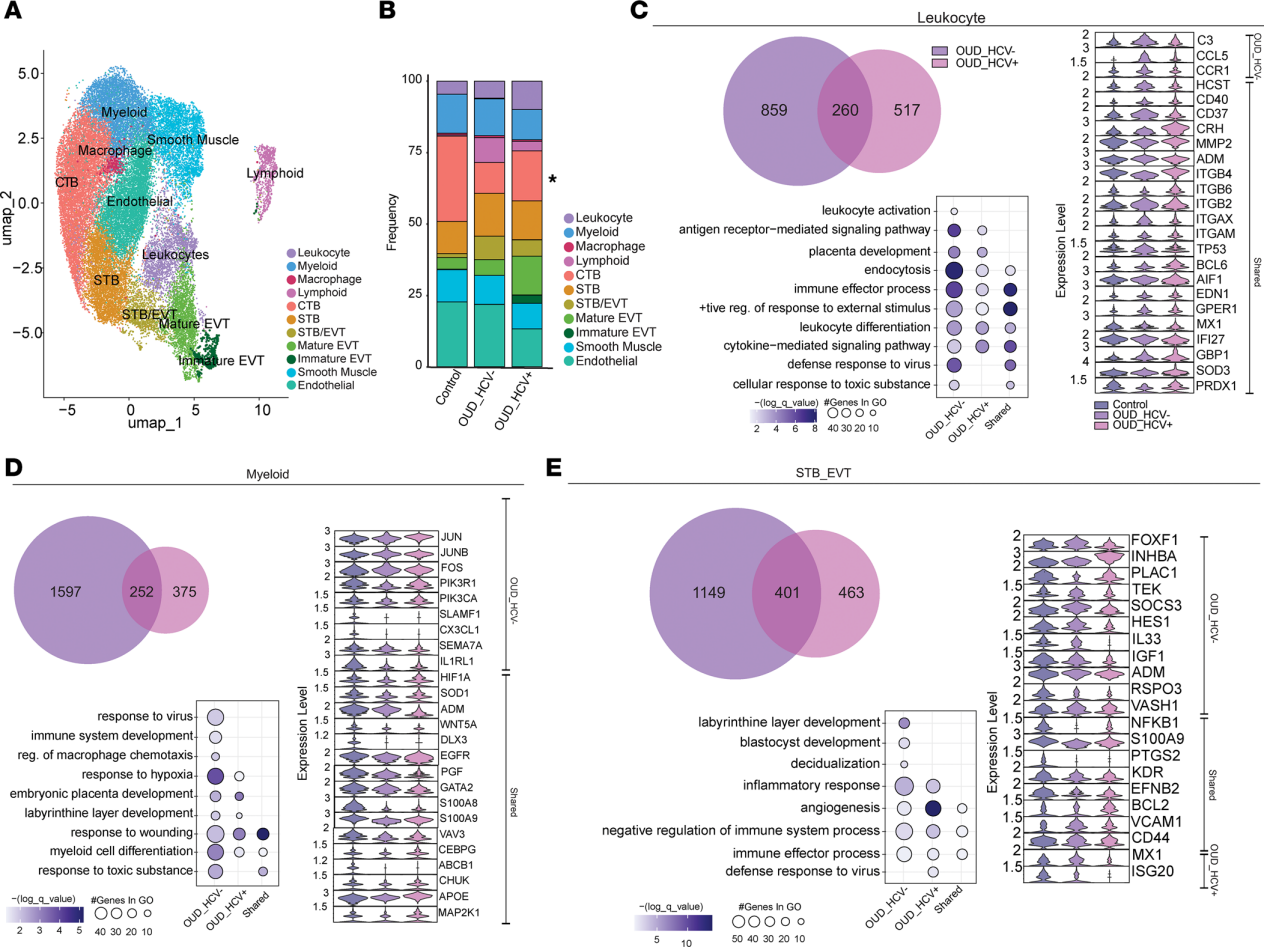
Maternal OUD±HCV alters the placental transcriptional profile on a spatial resolution. (**A**) UMAP of clusters from placental tissue isolated from control and OUD samples (36,009 spots). (**B**) Stacked bar graph of cluster frequencies (control 18,526 spots; OUD_HCV^–^ 9,837 spots; OUD_HCV^+^ 7,643 spots). Each cluster was assessed using 1-way ANOVA with Tukey’s HSD test. (**C**–**E**) Left: Venn diagram of DEG overlap from OUD_HCV^–^ versus control (purple) and OUD_HCV^+^ versus control (pink) comparisons. Middle: bubble plot of select GO terms from DEG enrichments using Metascape default settings. Size represents gene number and color represents log_10_ (*q* value). Right: violin plots of representative DEGs for (**C**) leukocyte, (**D**) myeloid, and (**E**) STB_EVT clusters. Control *n* = 2; OUD_HCV^–^ *n* = 1; OUD_HCV^+^ *n* = 1. *P < 0.05.

Given the impact of maternal OUD±HCV on placental structure and the transcriptional profiles of placental immune cells, we assessed gene expression changes in the context of placental architecture by Visium spatial transcriptomics (*n* = 2 control, *n* = 1 OUD_HCV^–^, *n* = 1 OUD_HCV^+^) (Figure 1A and Supplemental Figure 5, A and B). Uniform manifold approximation and projection (UMAP) revealed 11 subpopulations (Figure 5A, Supplemental Figure 5B, and Supplemental Table 2). Clusters were annotated using the EnrichR cell type database (Supplemental Figure 5C). We identified immune cells (leukocyte, myeloid, macrophage, and lymphoid); trophoblasts (cytotrophoblasts [CTBs], syncytiotrophoblasts [STBs], a combined STB/extravillous trophoblast [EVT] subset, and mature and immature EVT subsets), and structural cells (smooth muscle, endothelial) (Supplemental Figure 6C). Cluster frequencies were comparable between control and OUD groups, aside from a trending decrease in CTB abundance in the OUD groups (Figure 5B), a hallmark of placental dysfunction and stunted fetal growth (42).

Similar to the scRNA-Seq analyses, DEGs were identified from the Visium dataset and subjected to functional enrichment. The greatest number of DEGs was observed in the OUD_HCV^–^ group. DEGs within the leukocyte cluster mapped to shared biological processes regardless of maternal HCV status (Figure 5C). Genes important for immune effector functions, including antigen receptor–mediated signaling (*ITGAM/X/B2*, *HCST*, *CD40*, *CD37*), leukocyte differentiation (*BCL6*, *AIF1*), cytokine signaling (*EDN1*, *GPER1*), and antiviral responses (*ΜΧ1*, *IFI27*, *GBP1*) were upregulated in both OUD groups relative to the control (Figure 5C). Moreover, genes important for placental development (*CRH*, *MMP2*, *ADM*), endocytosis (*ITGB4/6*), and response to external stimuli (*TP53*), which were downregulated with OUD exposure alone, became upregulated with combined OUD and HCV exposure (Figure 5C). Additionally, genes linked to leukocyte activation (*C3*, *CCL5*, *CCR1*) were uniquely upregulated in the OUD_HCV^–^ group (Figure 5C). These findings suggest that maternal OUD alone is associated with heightened activation, antigen receptor signaling, and antiviral programs, whereas concomitant HCV infection shifts leukocyte function toward placental remodeling and stress response.

Within the myeloid cell cluster, shared upregulated DEGs in both OUD groups mapped to responses to toxic substances (*APOE*, *CHUK*, *MAP2K1*) and reduced expression of genes critical for placental homeostasis (Figure 5D). Specifically, genes associated with responses to hypoxia and wounding (*HIF1A*, *SOD1*, *ADM*, *S100A8/9*), myeloid cell differentiation (*VAV3*, *CEBPG*), and placental and labyrinthine layer development (*DLX3*, *EGFR*, *WNT5A*, *PGF*, *GATA2*) were all downregulated (Figure 5D). DEGs upregulated only in the OUD_HCV^–^ group also mapped to antiviral responses (*JUN*, *JUNB*, *FOS*); downregulated DEGs mapped to immune system development (*PIK3CA/R1*, *SLAMF1*) and regulation of macrophage migration (*CX3CL1*, *SEMA7A*, *IL1RL1*) (Figure 5D). These observations suggest a shift toward immune activation and away from tissue remodeling and placental maintenance.

Given that disrupted trophoblast function is associated with pregnancy complications, we next assessed DEGs within trophoblast clusters. CTBs serve as a progenitor population that continuously differentiate to replenish specialized trophoblast lineages at the syncytial surface within the extravillous space. Across both OUD groups, CTBs showed increased expression of genes involved in immune effector regulation (*SOCS3*, *IL6*, *JUN*) and hormone secretion (*NHBA*, *ADRA2A*) relative to controls (Supplemental Figure 5D). However, genes that mapped to positive regulation of cell motility and apoptosis (*ACTA2*, *MAPK14*, *AQP1*, *FAS*, *PAWR*), leukocyte differentiation (*S100A8*, *LYZ*), placental development (*GCM1*, *PLAC1*), and responses to hypoxia/wounding (*HIF1A*, *PTEN*, *S100A11*) were downregulated (Supplemental Figure 5D). Distinct transcriptional changes were identified between the OUD_HCV^–^ and OUD_HCV^+^ groups. In OUD_HCV^–^ CTBs, the expression of genes important for viral responses (*DDX3X*, *TLR2*, *MIF*), immune and vascular development (*BMI1*, *EZH2*, *ARG2*, *FLT4*, *PDGFRA*), and regulation of cellular senescence (*CDK1*, *ARHGAP5*) was reduced (Supplemental Figure 5D). However, in OUD_HCV^+^ CTBs, there was a selective upregulation of cytokine regulatory genes (*IL6*, *EDN1*) (Supplemental Figure 5D). These findings indicate that OUD skews CTBs toward a more immunoregulatory phenotype, potentially limiting placental growth and repair capacity.

STBs play important roles in host defense in late pregnancy. Regardless of maternal HCV status, genes associated with wound healing (*MAP3K20*, *MAPK1*, *GABPA*) and responses to oxidative stress or toxic substances (*SOD1*, *BAX*, *TP53*, *PRKRA*, *RRP7A*, *MFN2*) were downregulated with OUD (Supplemental Figure 6A). Additionally, DEGs mapping to embryonic organ development (*BMP7*, *GCM1*, *PDGFRA*) and TGF-β signaling (*SOCS3*, *SOX18*, *NPHP3*) were upregulated in the OUD_HCV^–^ group but downregulated with HCV coinfection (Supplemental Figure 6A). Additional DEGs downregulated with maternal OUD mapped to blastocyst development (*TFAP2C*, *HLX*, *NBN*) (Supplemental Figure 6A). On the other hand, genes associated with inflammatory responses (*TLR3*, *TXK*, *LYN*, *IGHA1*), macrophage activation (*BCL2*, *TWIST1*, *FOLR1*), and immune effector processes (*JUNB*, *ADM*, *BCAR1*) were downregulated with OUD_HCV^+^ (Supplemental Figure 6A). These findings suggest that OUD±HCV disrupts the role of STBs in maintaining immune tolerance at the maternal-fetal interface in late pregnancy.

We identified a combined STB/EVT cluster with transcriptional signatures mapping to homeostasis and immunoregulation (Figure 5E). Upregulated DEGs mapped to negative regulation of immune processes (*BCL2*, *VCAM1*, *CD44*) and downregulated DEGs to inflammatory responses and effector functions (*NFKB1*, *S100A9*, *PTGS2*) as well as angiogenesis (*KDR*, *EFNB2*) (Figure 5E). However, many genes important for placental integrity and blastocyst development were distinctly upregulated in OUD alone (*INHBA*, *FOXF1*, *PLAC1*, *TEK*, *HES1*, *VASH1*, *IL33*, *IGF1*, *ADM*) (Figure 5E). Finally, the expression of genes associated with antiviral mechanisms were altered with OUD and HCV infection (MX1 upregulated; ISG20 downregulated). These observations suggest that STB/EVT cells are skewed toward more regulatory processes, possibly to aid in maintaining placental structure with OUD exposure.

Previous studies have characterized the differentiation of EVTs from proliferative immature progenitors to mature invasive cells that play critical roles in placental development (43, 44). In line with these studies, our analysis identified 2 EVT clusters delineated as immature and mature. Although no DEGs were identified in the EVT_immature cluster, DEGs upregulated among the EVT_mature subset with maternal OUD, regardless of HCV status, mapped to immune signaling (*TYROBP*, *CD40*, *ITGAM*) and positive regulation of immune effector processes (*FCGR3A*, *ITGB2*) (Supplemental Figure 6B). However, downregulated genes in OUD mapped to circulatory system processes (*KDR*, *HBA2*, *CDH5*) and responses to toxic substances (*FASN*, *AREG*, *DDB1*) (Supplemental Figure 6B). Additionally, genes that were upregulated in OUD alone, but downregulated with HCV infection, mapped to inflammatory and antiviral responses (*STAT1*, *IFI27*, *MX1*, *IFI44L*) (Supplemental Figure 6B). Finally, upregulated DEGs unique to OUD mapped to positive regulation of immune responses (*PRF1*, *NKG7*, *CXCL10*); genes associated with placental development (*GCM1*, *PLAC1*, *PECAM1*) were downregulated (Supplemental Figure 6B). These findings indicate that mature EVTs are poised toward heightened inflammatory signaling with reduced abilities for vascular remodeling and adaptation to low oxygen.

Placental structural cells support healthy placental development and influence trophoblast and immune cell function. Downregulated DEGs in the smooth muscle cluster that were shared by both OUD groups mapped to TGF-β signaling (*TGFB3*), angiogenesis (*LT4*, *ADM*), responses to toxic substances and hypoxia (*GDF15*, *PRDX6*, *SOD1*, *HMOX2*, *UCP2*), inflammatory responses (*S100A9*, *CD55*, F*AS*, *BMP7*), and regulation of smooth muscle cell proliferation (*PLAT*, *ITGB3*) (Supplemental Figure 6C). Upregulated DEGs unique to the OUD_HCV^–^ group mapped to cellular response to environmental stimulus (*FOS*, *HSPA1A*); unique downregulated DEGs mapped to placental development (*GCM1*, *PDGFD*), viral processes (*JAK2*, *CHUK*, *REST*, *ID2*), response to corticosteroids (*GADD45A*, *FOXO3*, *SRF*, *USP14*), and regulation of trophoblast and smooth muscle cell migration (*FN1*, *ITGAV*, *APOLD1*, *FBN2*, *ADIPOR2*). These findings suggest that smooth muscle cells with OUD exposure are poised toward dampened responses to external stimuli and regulatory signaling.

DEGs upregulated in the endothelial cell cluster that were shared among both OUD groups mapped to blood vessel development (*VEGFC*, *BMP4*, *EPHA2*) and cell adhesion (*VCAM1*, *CHN1*); genes mapping to responses to hormones and inflammation (*S100A8/9*, *PRDX2*, *ΚLF9*) were downregulated (Supplemental Figure 6D). Additionally, OUD alone was associated with decreased expression of genes linked to immune activation (*GBP1*, *ITGB3*, *LYZ*), apoptosis (*FAS*, *DAPK1*, *BIRC2*), and responses to hypoxia and viruses (*DDX3X*, *IFNGR1*, *JAK2*, *GDF15*, *HSPB1*) (Supplemental Figure 6D). These findings suggest that OUD promotes vessel wall remodeling, akin to atherosclerosis (45).

### Maternal OUD disrupts cell communication networks in the term placenta.

**Figure 6:**
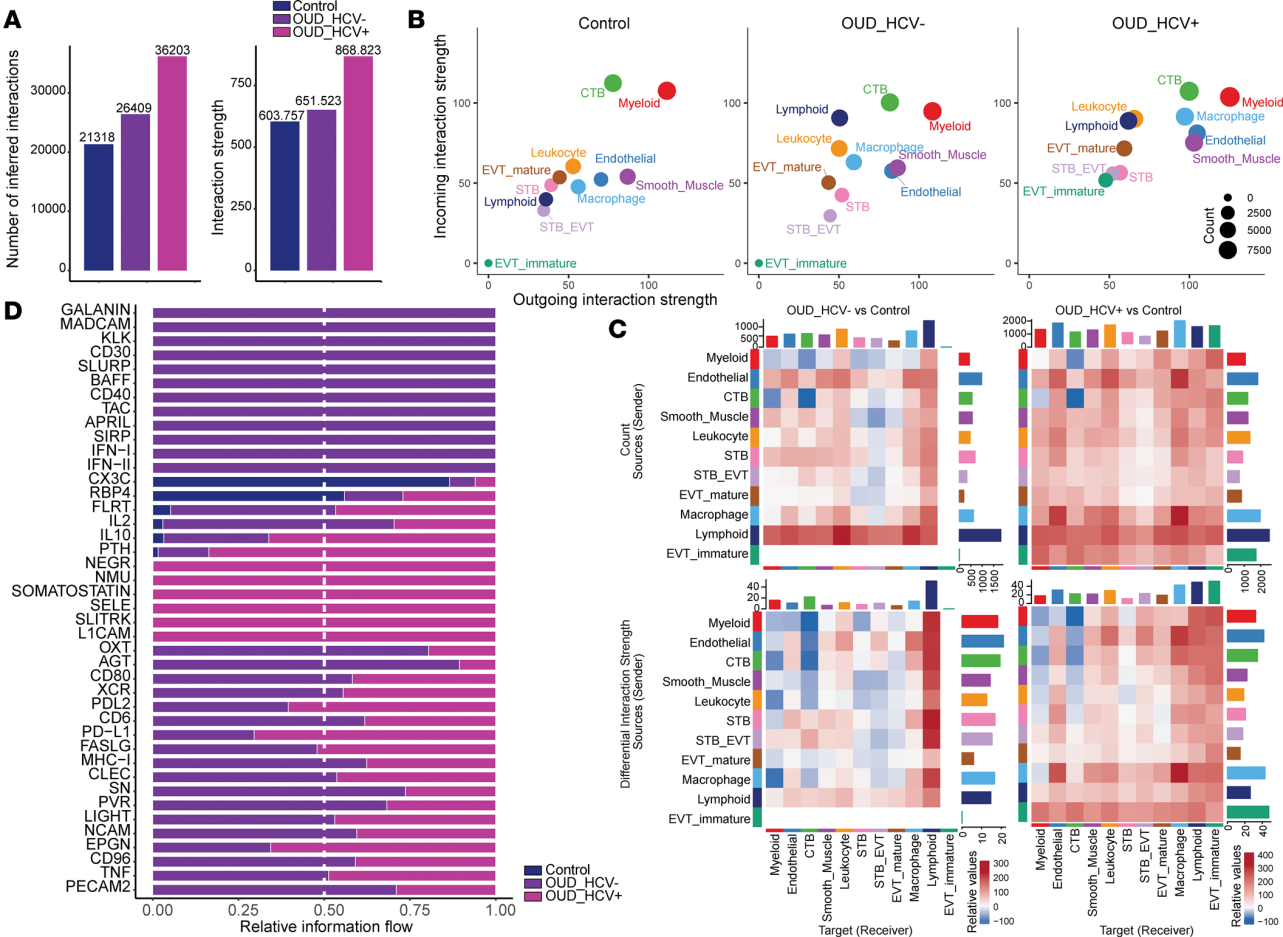
Maternal OUD±HCV affects communication networks in the placenta. (**A**) Bar plot of inferred interaction count (left) and strength (right). (**B**) Scatterplot of outgoing (*x* axis) and incoming (*y* axis) interaction strength for each cluster. Size indicates inferred interaction count. (**C**) Differential heatmap of interaction count (top) and strength (bottom) for OUD_HCV^–^ versus control (left) and OUD_HCV^+^ versus control (right). Top and side bar plots represent sum of interaction for the column and row, respectively. (**D**) Bar plot of relative information flow for indicated signaling pathways.

Given that many signaling pathways were dysregulated with maternal OUD±HCV, we employed CellChat to infer changes in cell-to-cell communication from the Visium spatial transcriptomics data (46). Overall communication patterns across the 11 placental cell populations revealed increased total ligand-receptor interactions and interaction strength in the OUD_HCV^+^ group, reflecting increased activation and cell recruitment (Figure 6A). We next compared the number and strength of outgoing and incoming signals of each subpopulation in control, OUD_HCV^–^, and OUD_HCV^+^ groups (Figure 6, B and C). The lymphoid cluster showed the strongest outgoing and incoming signaling with maternal OUD regardless of HCV status (Figure 6, B and C). Additionally, signaling toward trophoblast subsets was reduced, particularly in the OUD_HCV^–^ group (Figure 6, B and C). Moreover, EVT_immature signaling was only measurable in the OUD_HCV^+^ group (Figure 6, B and C), as this subset was not detected in the OUD_HCV^–^ group (Figure 5B). Therefore, maternal OUD augments placental lymphoid cellular communication while dampening inputs to trophoblast subsets.

To further delineate placental signaling pathways disrupted by maternal OUD±HCV, we compared information flow across control, OUD_HCV^–^, and OUD_HCV^+^ groups (Figure 6D). CX3C and RBP4 signaling was prominent in the control group but depleted with OUD (Figure 6D). CX3C, or fractalkine, has been shown to orchestrate placental leukocyte trafficking and activation, whereas RBP4 has been described as an important mediator of trophoblast function (47, 48). Overall CX3C signaling was mediated by myeloid and CTB clusters in the control group but was either depleted in the OUD_HCV^–^ group or shifted to leukocytes in the OUD_HCV^+^ group (Supplemental Figure 7, A and B). The RBP4 signaling axis was predicted to originate from lymphoid cells across groups, and it was strongest in the control group and depleted in both OUD groups (Supplemental Figure 7, A and C).

Additionally, we identified 12 signaling pathways unique to the OUD_HCV^–^ group (Figure 6D) important for cell trafficking (MADCAM [MAdCAM-1], TAC), lymphocyte activation and survival (BAFF, APRIL, CD40, CD30 [TNFSF8]), checkpoint signaling (SIRP, SLURP), vascular tone (GALANIN, KLK), and antiviral defenses (IFN-I, IFN-II) (Figure 6D). Overall signaling along these pathways was generally predicted to involve macrophage and lymphoid clusters (Supplemental Figure 7A). Notably, SIRP, CD40, and GALANIN signaling all originated from the lymphoid cluster targeting either all other cell subsets (SIRP, CD40) or macrophages (GALANIN) (Supplemental Figure 7A). Multiple signaling pathways originated from the macrophage subset, including CD30, MADCAM, SLURP, KLK, and IFN-I/II and targeted all other subsets (Supplemental Figure 7A). Finally, TAC signaling originated from immune and trophoblast subsets and was directed toward macrophages, whereas BAFF and APRIL signaling originated from all clusters and targeted the lymphoid cluster (Supplemental Figure 7A).

With HCV coexposure, 6 signaling pathways were identified, including neuropeptides that modulate trophoblast behavior and inflammation, neuromedin U (NMU), SLITRK, neuronal growth factor 1 (NEGR), and somatostatin, as well as adhesion molecules that regulate placental leukocyte trafficking E-selectin (SELE) and L1CAM (Figure 6D). The source of both NMU and NEGR signaling was macrophages and was received by CTBs or smooth muscle cells, respectively (Supplemental Figure 7A). Additionally, signaling along the SLITRK and SELE pathways primarily originated from myeloid cells and targeted all other cell types (Supplemental Figure 7A). Interestingly, the source of somatostatin and L1CAM signaling was lymphoid and EVT subsets and targeted either smooth muscle or all other clusters, respectively (Supplemental Figure 7A). Additionally, the EVT_immature cluster only identified in the OUD_HCV^+^ group was shown to be a hub for PTH, SLITRK, L1CAM, and EPGN signaling (Supplemental Figure 7A). PTH and EPGN signaling involving immature EVTs is thought to promote cell-cycle progression and motility while delaying terminal EVT differentiation (49, 50). These signaling patterns suggest that trophoblast-immune cell crosstalk is heightened with maternal HCV exposure, possibly to mediate immune tolerance and favor placental integrity.

## Discussion

The human placenta is crucial for supplying nutrients to and removing waste from the developing fetus (51). Maternal OUD can negatively affect placental structure via direct and indirect mechanisms. Opioids have been shown to target endogenous opioid receptors on trophoblast (52) and immune cells (53); however, the impact of these interactions on human placenta have not been well characterized (54, 55). Opioids can also indirectly affect placental function through disrupting the regulation of reproductive hormones, impairing placental blood flow, limiting trophoblast differentiation and invasion, and modulating inflammatory responses (10). Additionally, maternal HCV infection, commonly associated with intravenous drug use, can impair placental barrier structure (56). However, the impact of OUD±HCV on the maternal-fetal interface remains poorly understood.

Consistent with other reports of perinatal opioid (57) or HCV (58) exposure, newborns in our study were smaller, more likely to be admitted to an ICU, have NOWS, require pharmacological interventions (morphine), and be diagnosed with congenital abnormalities at a higher rate. These adverse outcomes are possibly mediated, in part, by abnormal placental development and therefore placental function throughout pregnancy (59, 60).

Our histological review per the Amsterdam criteria (61) revealed increased abundance of pathological findings in both OUD groups, notably features of FVM and villitis that are further exacerbated by HCV infection. Delayed villous maturation noted in opioid-exposed placenta was indicated by larger and less abundant terminal villi, restricting nutrient and oxygen exchange between mother and fetus (62). Fetal hypoxia is indicated by increased incidence of chorangiosis, a hallmark of low-grade hypoxia in placental tissue (63), as well as decreased expression of genes regulating oxygen levels across leukocyte, trophoblast, and endothelial subsets with maternal OUD. The transcriptional landscape of the placenta showed increased expression of genes associated with wound healing and vasculature remodeling in endothelial cells with maternal OUD regardless of HCV infection. These changes could be underlying vascular malperfusion prevalent in this cohort. Indeed, chronic inflammation is strongly associated with FVM and vessel wall thickening (64). In addition to maintaining the placental barrier, placental structural cells interact with surrounding trophoblasts and immune cells to mediate antimicrobial responses, control inflammation, and maintain fetal tolerance (65). However, gene expression signatures across smooth muscle and endothelial cell clusters suggest that OUD disrupts immune cell recruitment and antimicrobial responses. Furthermore, placental endothelial and trophoblast cells showed elevated GALANIN and KLK signaling to placental immune cells with maternal OUD alone. Signaling along the GALANIN and KLK pathways is linked to regulatory macrophage function, trophoblast survival, and placental vasculature (66, 67), providing a possible mechanism underlying increased placental pathologies with maternal OUD.

The Visium spatial transcriptomics analysis revealed a depletion of CTBs with OUD, which has been associated with placental hypoxia and reduced spiral artery remodeling, hallmarks of placental vascular malperfusion (42). Furthermore, we show that CTB transcriptional signatures shift away from tissue remodeling and placental repair with maternal OUD, regardless of HCV status, as shown by decreased expression of genes important for placental development and responses to wounding or hypoxia. Analysis of cell communication networks further revealed dampened signaling to trophoblast subsets, possibly contributing to poor maintenance of the placental barrier and immune function (14). These structural changes have substantial implications for fetal health since delayed villous maturation has been recently identified as predictor of low scores on neurodevelopmental tests, intrauterine growth restriction, and congenital abnormalities, all of which are increased with maternal OUD (68). Indeed, increased placental signaling along multiple pathways involved in neurodevelopment and behavior with OUD and HCV coexposure offer possible mechanisms underlying poor neurodevelopmental outcomes in newborns.

Lack of placental RBP4 signaling has been associated with aberrant angiogenesis and enlarged maternal vascular spaces (69). A loss of RBP4 signaling from lymphoid cells to trophoblasts was prominent in both OUD groups, in line with our findings of increased incidence of MVM with maternal OUD. In addition, transcriptional profiles of CTBs and STBs were indicative of barrier dysfunction and defects in wound healing, and the transcriptional signatures of EVT_mature suggested heightened immune and inflammatory signaling but reduced vascular remodeling and antioxidant processes, akin to insufficient trophoblast invasion (70). Insufficient trophoblast differentiation/function is associated with prominent placental pathologies that have been associated with opioid use during pregnancy, including fetal growth restriction, placental abruption, and preterm birth (43).

We report elevated concentrations of proinflammatory factors with maternal OUD in the decidua. Although a proinflammatory milieu is important for initiating labor and parturition in late pregnancy, exacerbated markers of inflammation are noted in several complications, including preeclampsia and recurrent miscarriage (71). Notably, elevated levels of decidual TNF impair trophoblast recruitment and function as well as the expression of angiogenesis factors (72). Dysregulated cytokine secretion with maternal OUD is less profound in the (villous) fetal compartment. Low concentrations of IL-6 and IL-8 impede trophoblast migration and angiogenesis and have been associated with impaired fetal development and immune-endocrine crosstalk (73). On the other hand, elevated levels of IL-12 and IL-7 have been associated with fetal growth restriction (74), in line with small-for-gestational-age newborns and vascular malperfusion and increased rates of gestational hypertension with maternal OUD. Furthermore, these findings align with dysregulated expression of genes important for repairing tissue damage and responding to hypoxia/growth factors across most cell subsets identified by our spatial transcriptomics analysis.

Among immune cell populations, we report a decreased frequency of the regulatory dMac_3 macrophage subset with maternal OUD and HCV infection by flow cytometry. Given their regulatory functions, the depletion of dMac_3 cells with maternal OUD could explain elevated concentrations of inflammatory markers and impaired tissue-repair functions. Moreover, loss of regulatory macrophages has been linked to placental insufficiency (75). These changes in immune cell frequency detected by scRNA-Seq with maternal OUD±HCV were not as prominent as those measured by flow cytometry. This discrepancy may be attributed to the fact that protein markers can remain stable despite fluctuations in transcriptional profiles (76). Nevertheless, scRNA-Seq analysis of decidual macrophage subsets showed upregulation of processes associated with immune activation and inflammatory responses across all subsets with maternal OUD. However, antimicrobial responses by decidual macrophages to ex vivo bacterial TLR stimulation were dampened with maternal OUD±HCV infection, suggesting an immune tolerant–like state. Furthermore, transcriptional profiles of PAMM1B, PAMM1A, and PAMM2 cells were characterized by heightened expression of tissue repair and placental development mechanisms. Activated but functionally defective decidual macrophages have been associated with multiple placental pathologies, including villitis, vascular malperfusion, disrupted cytokine/chemokine signaling networks, and trophoblast differentiation (77), all of which align with our findings with maternal OUD.

Maternal monocytes and macrophages contribute to maintaining immune tolerance, pathogen defenses, and labor onset through interactions with decidual T cells (78). We report dampened CD4^+^ and CD8^+^ T cell functional responses to stimulation with maternal OUD±HCV, suggesting an immune tolerant or exhausted T cell phenotype. This was further reflected by our scRNA-Seq data, where expression of genes important for immune responses and cell killing were dampened with maternal OUD. Additionally, our findings indicate decreased abundance of CD56^+^CD16^+^ dNK cells, a unique subset of dNK cells that are poor cytokine producers but are highly cytotoxic, important for responding to infection and regulating trophoblast invasion (79). Here, our data showed decreased abundance and poor degranulation capacity of CD56^+^CD16^+^ dNK cells with maternal OUD±HCV.

In summary, this study used multiomic approaches to uncover the impact of OUD±HCV on placental structure and immune function. Our findings shed light on the complex interplay between endothelial, smooth muscle, trophoblast, and immune cells, providing a framework for possible mechanisms underlying poor placental development with maternal inflammatory conditions. Maternal OUD, particularly when combined with HCV infection, profoundly alters the immune and structural landscape of the placenta, driving adverse pregnancy and neonatal outcomes through chronic inflammation, immune dysregulation, and impaired placental vascularization. However, this study does present some limitations. Notably, we were unable to stratify by additional maternal factors without compromising statistical power. Parity was significantly higher in both OUD groups compared with the control group. Additionally, polysubstance use is prevalent with people who use drugs, including high rates of cannabis, nicotine, alcohol, and stimulant use, and reliance on self-reported substance use histories introduces potential recall and social desirability bias that may limit accurate reporting. Future larger cohort studies should better control for these confounding variables.

## Methods

### Sex as a biological variable.

Only pregnant women were included in this study; fetal sex was not considered as a biological variable.

### Sample collection.

Placental biopsies were collected from full-term pregnancies (metadata provided in Table 1). Leukocytes from decidua basalis and chorionic villous tissues were isolated as previously described (19, 80). Additional placental tissue segments were flash-frozen for homogenization. A third piece of tissue was fixed in 10% formalin and paraffin embedded for histology. PBMCs and plasma were isolated from maternal blood samples collected at delivery as previously described (19).

### HCV IgG and IgM ELISAs.

HCV IgG and IgM antibodies were detected in maternal plasma using an indirect ELISA. Plates were coated with 1 μg/mL HCV combined recombinant antigen (RayBiotech, 228-10628) in PBS overnight at 4°C. Plates were blocked with 200 μL/well blocking buffer (BB) for 1 hour at room temperature. Maternal plasma was heat-inactivated for 30 minutes at 55°C, added to duplicate wells in BB (1:100 for IgG, 1:50 for IgM), and incubated for 1 hour at room temperature. HRP–anti-human IgG or IgM (BD Pharmingen, 1:4,000 in BB) was added and incubated for 1 hour at room temperature. The reaction was visualized using *o*-phenylenediamine/30% H_2_O_2_ and stopped after 20 minutes with the addition of 1 M HCl. The OD was read at 490 nm absorbance on the SpectroMax iD3 plate reader (Molecular Devices). Positive and negative plasma controls were used to determine suitable cutoff values for IgG and IgM (HCV IgG OD > 0.096, HCV IgM OD > 0.408).

### HCV viral load detection by RT-PCR.

RNA was extracted from HCV-IgG– or HCV-IgM–positive maternal plasma using the Quick-RNA Viral 96 kit (Zymo Research, R1041), reverse-transcribed using the High-Capacity cDNA Reverse Transcription kit (Applied Biosystems, 4368814) on the Nexus Gradient MasterCycler (Eppendorf). Reverse transcription cycling conditions were the following: 10 minutes at 25°C, 120 minutes at 37°C, and 5 minutes at 85°C with a 4°C hold. Viral cDNA was amplified with HCV 5′ UTR/partial core forward and reverse primers (HCV_5UTR_1_F: 5′-GTCTAGCCATGGCGTTAGTATGAGTG-3′ and HCV_5UTR_1_R: 5′-ACAAGTAAACTCCACCAACGAG-3′) using Platinum SuperFi II PCR Master Mix (Invitrogen, 12368010). PCR conditions were the following: initial denaturation 98°C for 10 seconds; 35 cycles of denaturation (98°C for 10 seconds), annealing (60°C for 10 seconds), and extension (72°C for 30 seconds); and a final extension at 72°C for 5 minutes. Products were run on 1.5% agarose gels alongside extraction, reverse-transcribed, and PCR-negative controls in triplicate and imaged with GelRed Nucleic Acid Stain (Biotium, 41003) using the Azure 300 Imager (Azure Biosystems). Samples were considered positive for HCV viral RNA if an amplification product corresponding to 374 bp was detected.

### Assessment of placental structure and histology.

Placental histology was assessed by review of H&E-stained placental tissues. Placental lesions were documented and classified per the Amsterdam Consensus Statement Guidelines (61) by a placental pathologist at the University of Kentucky College of Medicine.

### Luminex assays.

Flash-frozen placental tissues were homogenized as previously described (40) for Luminex assay per the manufacturer’s instructions using the R&D Systems Human Luminex Discovery assays, LXSAH (inflammatory) and LXSAHM (angiogenesis). Markers of placental function/development in maternal circulation were measured in maternal plasma using the R&D Systems Human Luminex Discovery assay (LXSAHM, angiogenesis), PAPP-A Human ProcartaPlex Simplex kit (EPX010-12393-901), and MilliporeSigma Human Angiogenesis and Growth Factor panel (HAGP1MAG-12K). sPLSDA from Luminex data was performed using the MixOmics (81) package with normalized counts.

### Phenotyping by flow cytometry.

Placental leukocytes were stained with the following cocktails for 20 minutes at 4°C: for decidua, CD45 (FITC, clone: HI30, RRID: AB_314394), CD4 (BV510, clone: RPA-T4, RRID: AB_2563313), CD8 (ECD, clone: SK1, RRID: AB_1953243), CD14 (AF700, clone: M5E2, RRID: AB_493747), HLA-DR (APC-Cy7, clone: L243, RRID: AB_314682), CD11c (PerCP-Cy5.5, clone: 3.9, RRID: AB_10640733), CD56 (BV711, clone: HCD56, RRID: AB_11218788), CD16 (PB, clone: 3G8, RRID: AB_2104003), FOLR2 (PE, clone: 94b/FOLR2, RRID: AB_2721335), S100A8/9 (APC, clone: 27E10, RRID: AB_1227536), and CD9 (PE-Cy7, clone: HI9A, RRID: AB_2728256); for chorionic villous, CD45 (BV510, clone: HI30, RRID: AB_2561942), CD14 (AF700, clone: M5E2, RRID: AB_493747), HLA-DR (APC-Cy7, clone: L243, RRID:AB_314682), FOLR2 (PE, clone: 94b/FOLR2, RRID: AB_2721335), CD9 (PE-Cy7, clone: HI9A, RRID: AB_10562251), and CCR2 (BV605, clone: K036C2, RRID: AB_2563980) (all from Biolegend).

True-Stain Monocyte Block and Human TruStain FcX (BioLegend) were added to the surface staining cocktail (1:20). Cells were washed, run on the Attune NxT, and analyzed on FlowJo 10.10 (Becton Dickinson). Representative gating strategies are shown in Supplemental Figure 1D and Supplemental Figure 3D.

### Ex vivo stimulation and intracellular cytokine staining.

First, 1 × 10^6^ leukocytes were stimulated for 16 hours at 37°C in 10% FBS/RPMI with or without bacterial TLR cocktail containing 1 mg/mL LPS-B5 (TLR4, tlrl-b5lps, InvivoGen), 2 mg/mL Pam3CSK4 (TLR1/2, TLRL-PMS, InvivoGen), and 1 mg/mL FSL-1 (TLR2/6, SML1420, Sigma-Aldrich). Brefeldin A (Biolegend) was added 1 hour after initial stimulation.

Decidual leukocytes were stained with CD45 (FITC, clone: HI30, RRID: AB_314394), CD2 (BV510, clone: RPA-2.10, RRID: AB_2566040), CD20 (BV510, clone: 2H7, RRID: AB_2563237), CD14 (AF700, clone: M5E2, RRID: AB_493747), HLA-DR (APC-Cy7, clone: L243, RRID: AB_314682), CD11c (PE-eF610, clone: 3.9, RRID: AB_2574532, Thermo Fisher), CD9 (PE-Cy7, clone: HI9A, RRID:AB_2728256), and CCR2 (BV605, clone: K036C2, RRID: AB_2563876) (all from BioLegend except cd11c, ThermoFisher). Chorionic villous leukocytes were stained with CD45 (BV510, clone: 2D1, RRID: AB_2687377), CD14 (AF700, clone: M5E2, RRID: AB_493747), HLA-DR (APC-Cy7, clone: L243, RRID: AB_314682), FOLR2 (PE, clone: 94b/FOLR2, RRID: AB_2721335), CD9 (PE-Cy7, clone: HI9A, RRID: AB_2728256), and CCR2 (BV605, clone: K036C2, RRID: AB_2563980) (all from Biolegend). Surface stains were incubated for 30 minutes in the dark at 4°C. Samples were fixed and permeabilized using BioLegend FixPerm (catalog 420801) at 4°C for 20 minutes and stained intracellularly for 4 hours for TNF-α (APC, clone: MAb11, RRID: AB_315261), IL-6 (PerCP, clone: MQ2-13A5, RRID: AB_1115151, ThermoFisher), IL-1β (FITC, clone: JK1B-1, RRID:AB_345362), and MIP-1β (PE, clone: D21-1351, RRID: AB_2562770) (all from BioLegend except IL-6, ThermoFisher). Samples were washed, acquired, and analyzed as outlined for phenotyping. Representative gating strategies are shown in Supplemental Figure 2A.

First, 1 × 10^6^ decidual leukocytes were stimulated for 16 hours at 37°C in 10% FBS/RPMI in the presence or absence of PMA/ionomycin. CD107a (PB, clone: H4A3, RRID: AB_2265606) was added during the stimulation. Brefeldin A was added 1 hour after initial stimulation. Decidual leukocytes were stained with CD45 (PerCpCy5.5, clone: HI30, RRID: AB_893340), CD4 (PB, clone: OKT4, RRID:AB_1595438), CD8 (ECD, clone: SK1, RRID: AB_1953243), HLA-DR (APC-Cy7, clone: L243, RRID: AB_314682), and CD56 (BV711, clone: 5.1H11, RRID:AB_2565920) for 30 minutes at 4°C. Samples were fixed, permeabilized, and stained intracellularly for IL-2 (AF700, clone: MQ1-17H12, RRID: AB_528929), IL-17 (FITC, clone: BL168, RRID: AB_961390), TNF-α (APC, clone: MAb11, RRID: AB_315261), and IFN-γ (PE-Cy7, clone: 4S.B3, RRID: AB_2123323) (all from Biolegend) for 4 hours at 4°C. Cells were then washed, acquired, and analyzed as outlined for phenotyping. Representative gating strategies are shown in Supplemental Figure 2, B and C.

### Phagocytosis assay.

Leukocytes were incubated for 2 hours at 37°C in media containing 1 mg/mL pH-sensitive pHrodo *E*. *coli* BioParticles conjugates (Thermo Fisher Scientific). Cells were stained with CD45 (BV510, clone: 2D1, RRID: AB_2687377), CD14 (AF700, clone: M5E2, RRID: AB_493747), HLA-DR (APC-Cy7, clone: L243, RRID: AB_314682), FOLR2 (PE, clone: 94b/FOLR2, RRID: AB_2721335), CD9 (PE-Cy7, clone: HI9A, RRID: AB_2728256), and CCR2 (BV605, clone: K036C2, RRID: AB_2563876) (all from Biolegend), and then resuspended in ice-cold FACS buffer. Cells were washed, acquired, and analyzed as outlined for phenotyping. A pHrodo-negative control (cells incubated without pHrodo-labeled particles) was used to establish background fluorescence and gating thresholds. A representative gating strategy is shown in Supplemental Figure 2D.

### CD45 3′ single-cell RNA library preparation.

Placental leukocytes were stained with CD45 (FITC, clone: HI30, RRID: AB_314394) for 20 minutes at 4°C in 1% FBS/DPBS and sorted into 30% FBS/RPMI using the BD FACSAria III cell sorter. Cells were counted in triplicate, and an equivalent number of cells were pooled by group before resuspension in 0.4% BSA/PBS to a final concentration of 1,200 cells/μL and immediately loaded on the 10x Genomics Chromium Controller with a loading target of 30,000 cells. Libraries were generated per the manufacturer’s instructions (V3.1, 10x Genomics). Libraries were sequenced using the Illumina NovaSeq 6000 with a sequencing target of 20,000 reads/cell.

### scRNA-Seq analysis.

Raw reads were aligned and quantified using the Cell Ranger software suite (v6.0.1, 10x Genomics) against the GRCh38 human reference genome. Downstream processing of aligned reads and quality control was performed using Seurat (v5.1.0), as previously described (19). Data objects for the placenta were integrated with data from peripheral PBMCs to remove infiltrating leukocytes, as previously described (19). Data normalization, dimensional reduction, cell type assignment (Supplemental Table 1), and differential gene expression analysis (Supplemental Table 1) were performed as previously described (19).

### Visium spatial transcriptomics library preparation.

Placental tissues without any identified pathological abnormalities were assessed by Visium spatial transcriptomics (10x Genomics) with CytAssist using Demonstrated Protocol CG000520. RNA was extracted using QIAGEN FFPE RNA kit per the manufacturer’s protocol. Blocks with a DV200 greater than 40% were deparaffinized and H&E stained. Imaging was performed on a Nikon Ni-E microscope and image tiles stitched with Nikon Elements software. Slides were decrosslinked and immediately hybridized with the Visium Human Transcriptome Probe kit V2 (PN-1000466, 10x Genomics) per the manufacturer’s instructions. Libraries were sequenced using the Illumina NovaSeq 6000 with a sequencing target of 25,000 paired reads per covered spot.

### Visium spatial transcriptomics analysis.

Raw reads were aligned and quantified using Space Ranger software suite (v3.1.1, 10x Genomics) against the GRCh38 human reference genome and Human Transcriptome Probe set v2. Downstream processing of reads and initial quality control was performed using Seurat (v5.1.0). Data normalization, dimensional reduction, cell type assignment (Supplemental Table 2), and differential gene expression analysis (Supplemental Table 2) were also performed as previously described (19).

### Cell-cell interaction analysis.

A CellChat (46) object was generated from the Seurat object with the createCellChat function. Data were preprocessed with identifyOverExpressedGenes and identifyOverExpressedInteractions functions before communication probabilities were determined with computeCommunProb (truncatedMean, trim = 0.1, interaction, range = 250, contact.range = 100). Communications were filtered to a minimum number of 10 cells, and signaling pathway probabilities were calculated.

### Statistics.

Data analysis from Luminex and flow cytometry assays were performed as follows. Normality was assessed using the Shapiro-Wilk test (α = 0.05) and outliers identified via ROUT analysis (*q* = 0.1%). For normally distributed datasets, group differences were evaluated using unpaired *t* tests (2-tailed) with Welch’s correction (2 groups) or 1-way ANOVA (3 groups). For non-Gaussian data, Mann-Whitney (2 groups) or Kruskal-Wallis (3 groups) tests were applied. For 3-way group comparisons by ANOVA, post hoc pairwise comparisons were conducted using Tukey’s honestly significant difference (HSD) test. Tukey’s HSD was selected as it provides strong control of the family-wise error rate and is robust to moderate differences in group sample sizes. All possible pairwise group comparisons (control vs. OUD_HCV^–^, control vs. OUD_HCV^+^, OUD_HCV^–^ vs. OUD_HCV^+^) were assessed, and adjusted *P* values were reported. Categorical data were compared using χ^2^ or Fisher’s exact tests as appropriate. Statistical significance was set at *P* less than 0.05, after adjustment, with results trending toward significance noted at *P* less than 0.1. We report adjusted (*q* value) and unadjusted *P* values throughout to facilitate interpretation. All statistical analyses were performed using GraphPad Prism and R (https://cran.r-project.org/). Unless otherwise noted, all error bars represent the SEM, symbols in black denote comparisons to the control, and symbols in blue denote direct comparison between the OUD_HCV^–^ and OUD_HCV^+^ groups. Limitations related to residual confounding from demographic and clinical covariates and potential impact on immunological findings are discussed further in the limitations section of the Discussion.

### Data availability.

The datasets supporting the conclusions of this article are available on NCBI’s Sequence Read Archive: scRNA-Seq (decidua: PRJNA1234184, villous: PRJNA1234178) and Visium spatial transcriptomics (PRJNA1231418). Values for all data points in graphs are reported in the Supporting Data Values file.

## Author contributions

IM, JMB, and CC were responsible for conceptualization. IM, JMB, and CC contributed to methodology. HET, BMD, SBW, NRS, and MEB conducted the investigation. HET, BMD, SBW, DCM, NRS, and IM wrote the manuscript. HET and IM acquired funding. CC and JMB enrolled participants. All authors have read and approved the final draft of the manuscript.

## Conflict of interest

The authors have declared that no conflict of interest exists.

## Funding support

This work is the result of NIH funding, in whole or in part, and is subject to the NIH Public Access Policy. Through acceptance of this federal funding, the NIH has been given a right to make the work publicly available in PubMed Central. The content is solely the responsibility of the authors and does not necessarily represent the official views of the NIH or the University of Kentucky.

- NIH grants 1R01DA059152-01 (to IM and JOB), 7R01AI145910-05S1 (to IM), TL1TR001997 (to HT), UL1TR001998 (to University of Kentucky Center for Clinical and Translational Science), and P30CA177558 (to the University of Kentucky Biospecimen Procurement & Translational Pathology Shared Resource Facility of the Markey Cancer Center).
- University of Kentucky Clinical and Translational Science Substance Use Disorder pilot grant 3210003238 (to IM and JOB).
- Kentucky Opioid Response Effort (KORE) via Substance Abuse and Mental Health Services Administration (SAMHSA) grants H79TI081704 and H79TI083283 (to the Kentucky Cabinet for Health and Family Services).

## Supplementary Material

Supplemental data

Supplemental table 1

Supplemental table 2

Supporting data values

## Figures and Tables

**Table 1:**
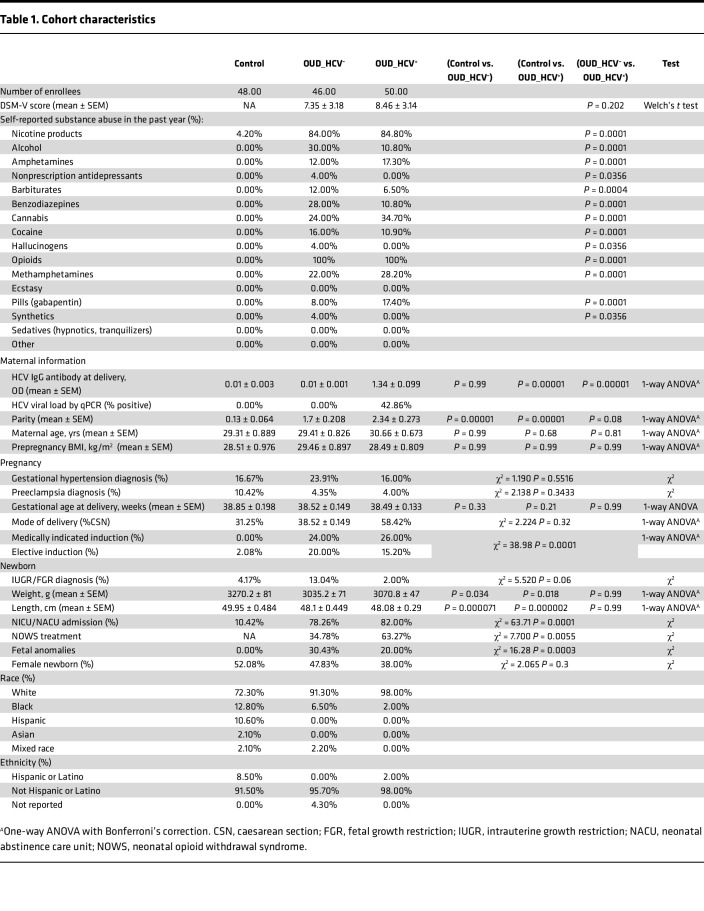
Cohort characteristics

## Version 1 03/17/2026

In-Press Preview

## Version 2 05/08/2026

Electronic publication
