# Supplementary material for: Maternal opioid use and hepatitis C infection disrupt the placental immune landscape and structure
Source: JCI Insight. 2026 Mar 17;11(9):e199606. doi: 10.1172/jci.insight.199606 (PMC13232015; doi:10.1172/jci.insight.199606)

Supplemental Figure 1

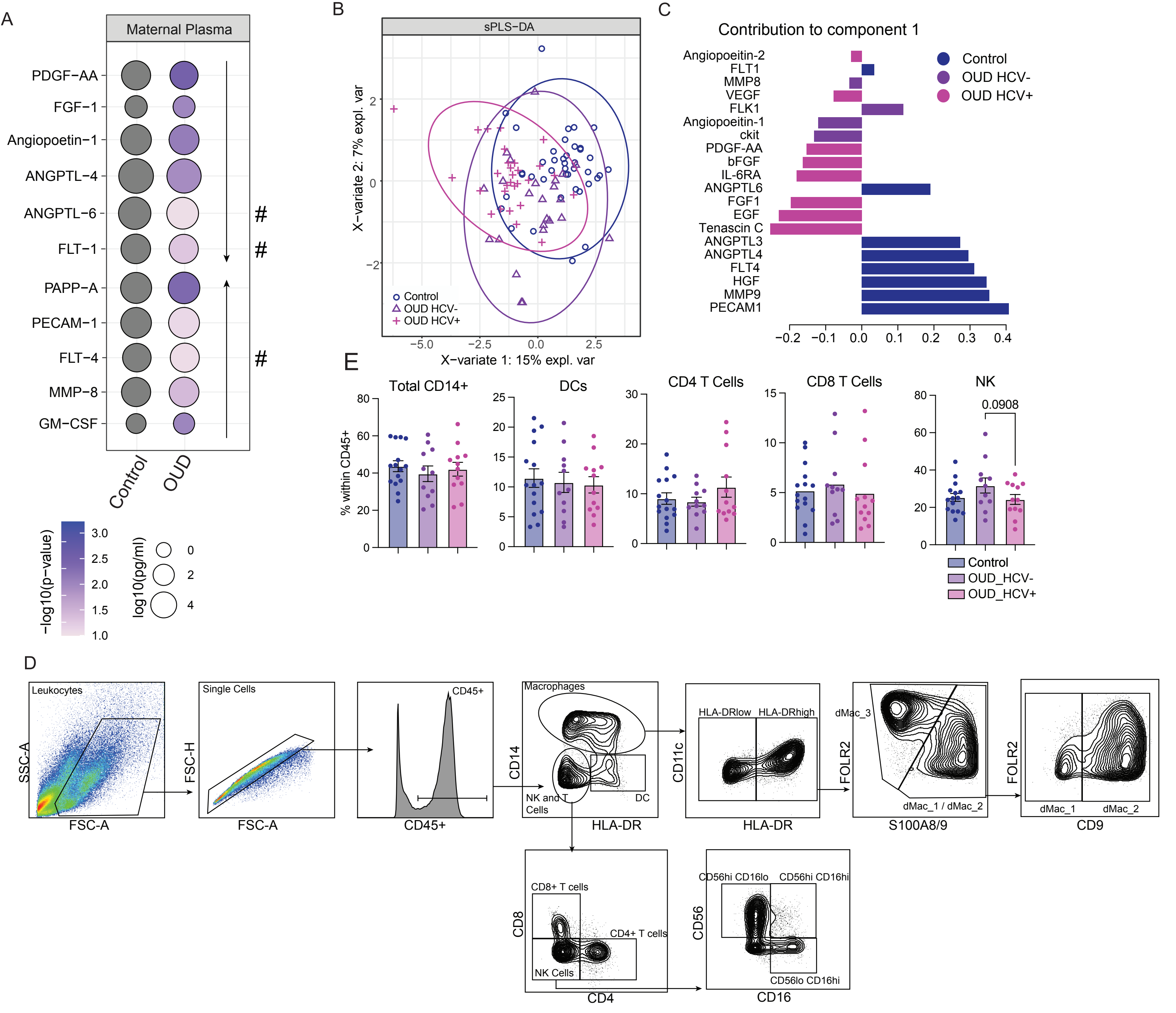

Supplemental Figure 2

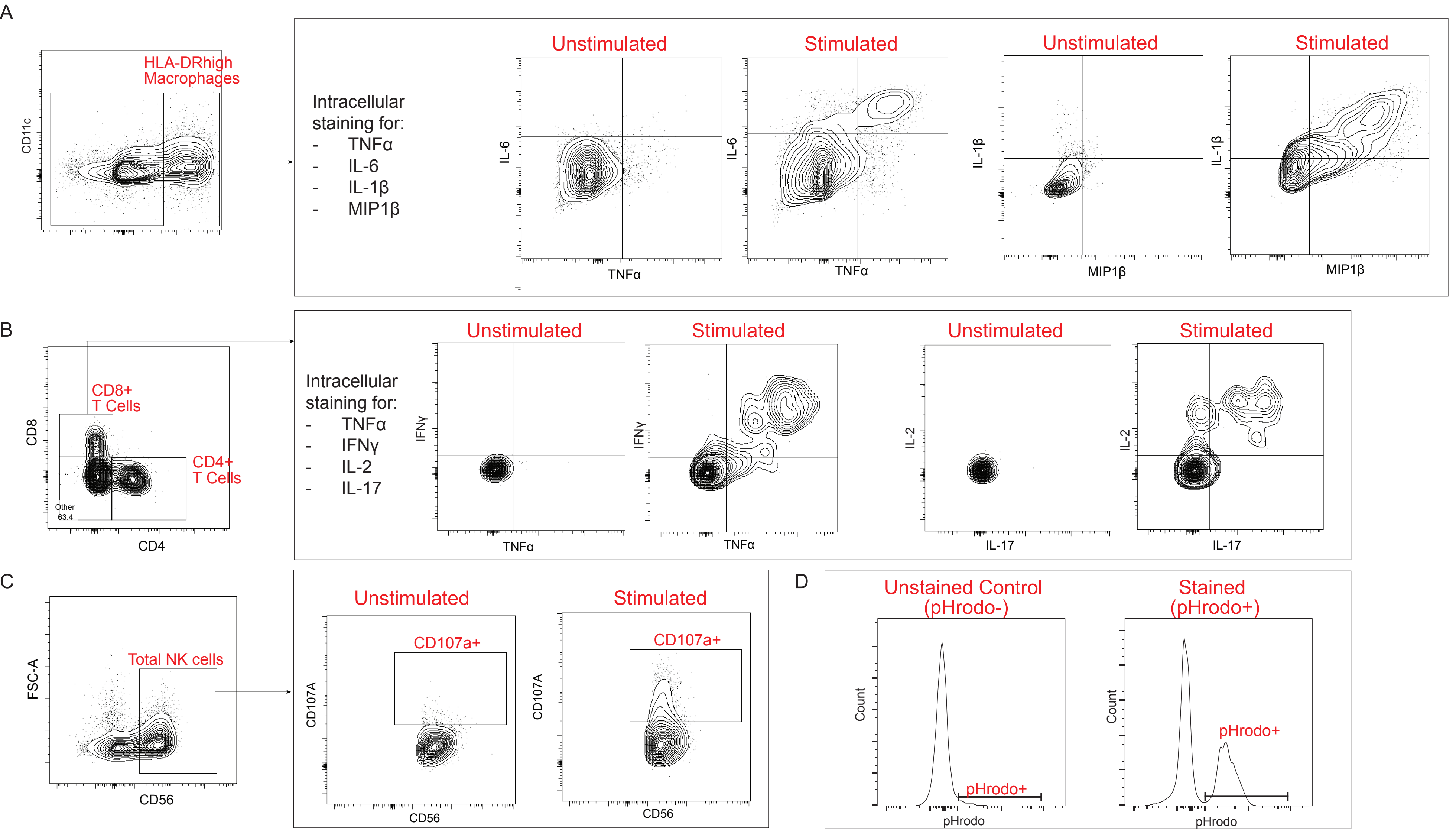

Supplemental Figure 3

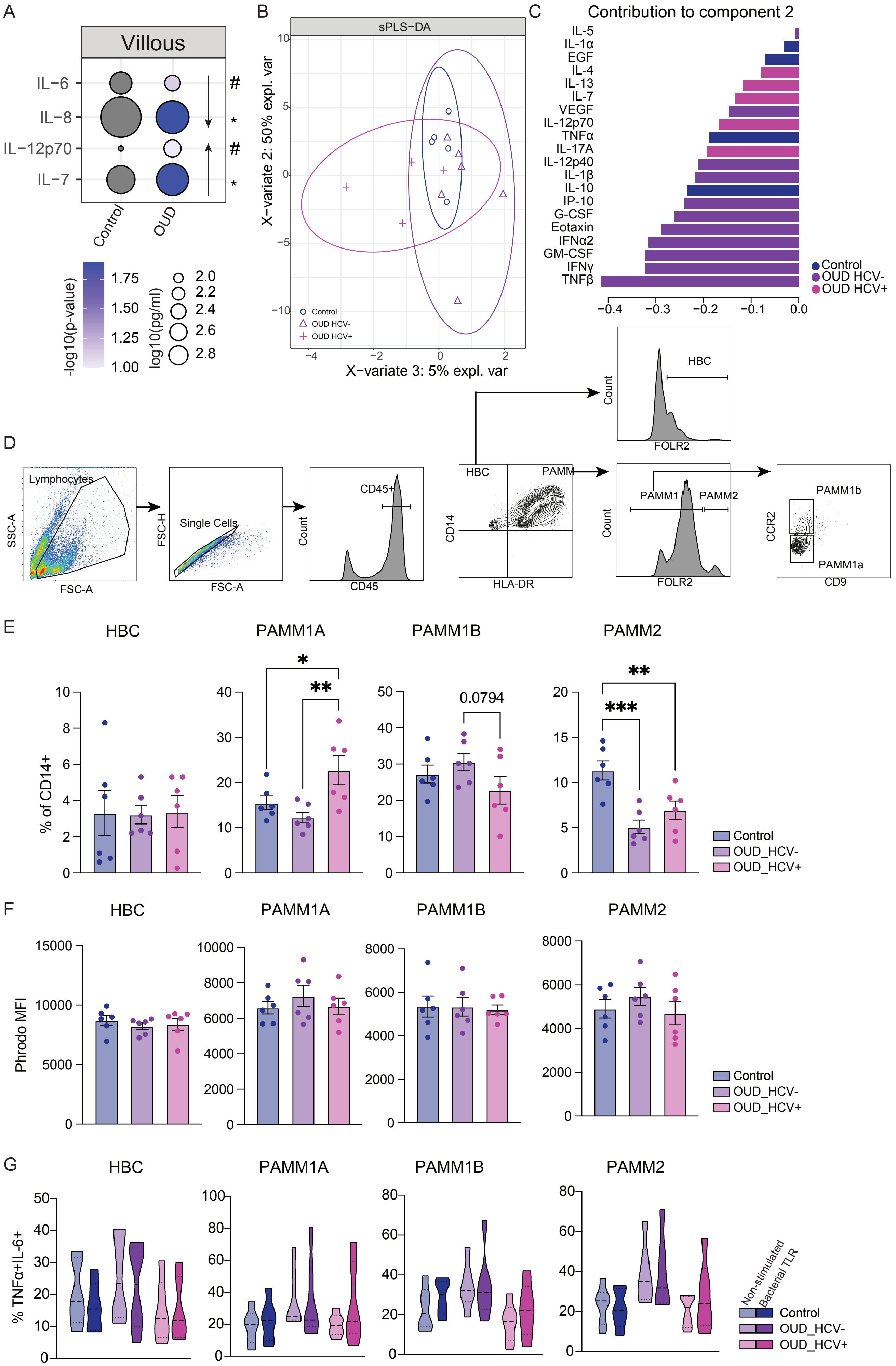

Supplemental Figure 4

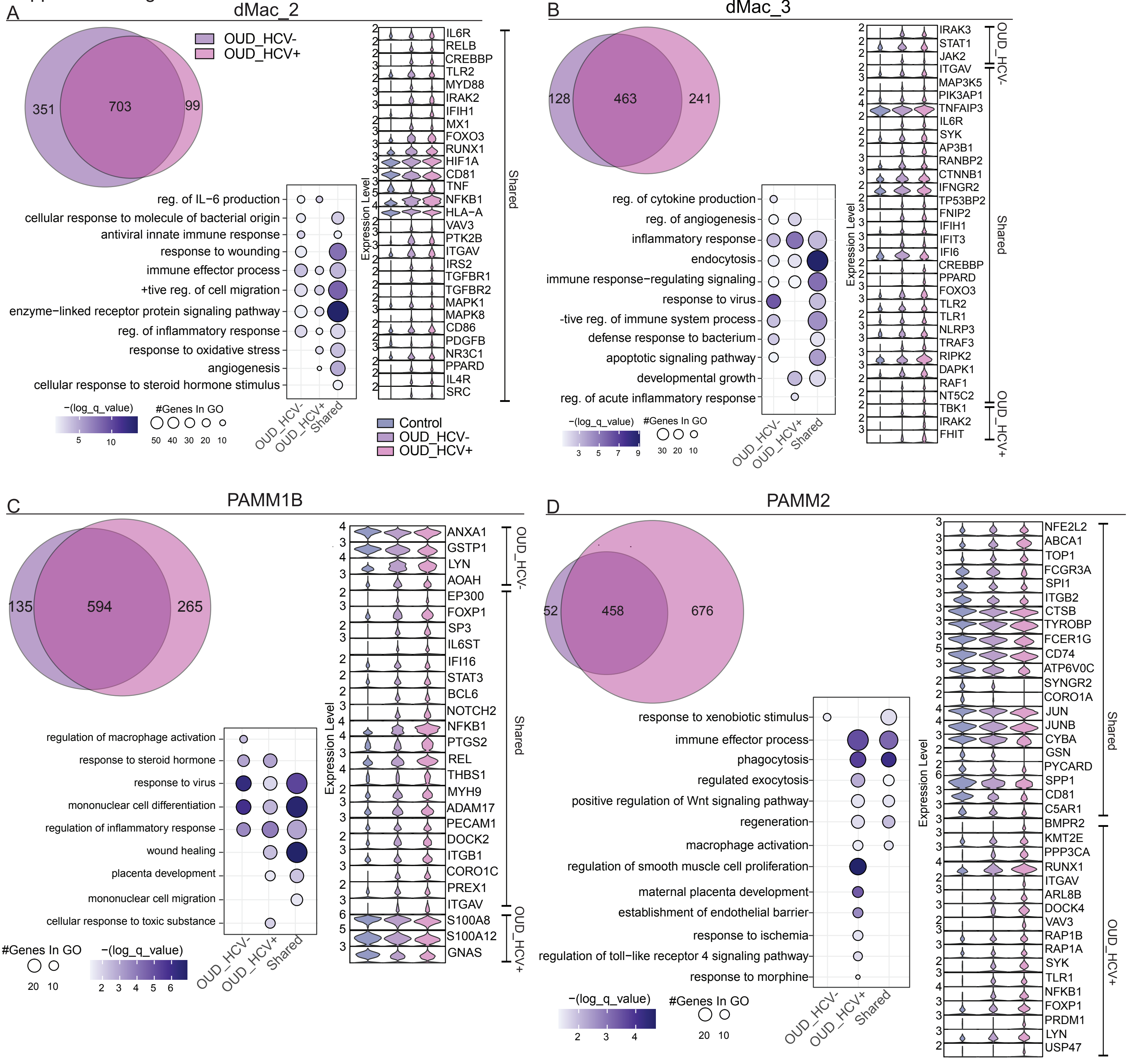

Supplemental Figure 5

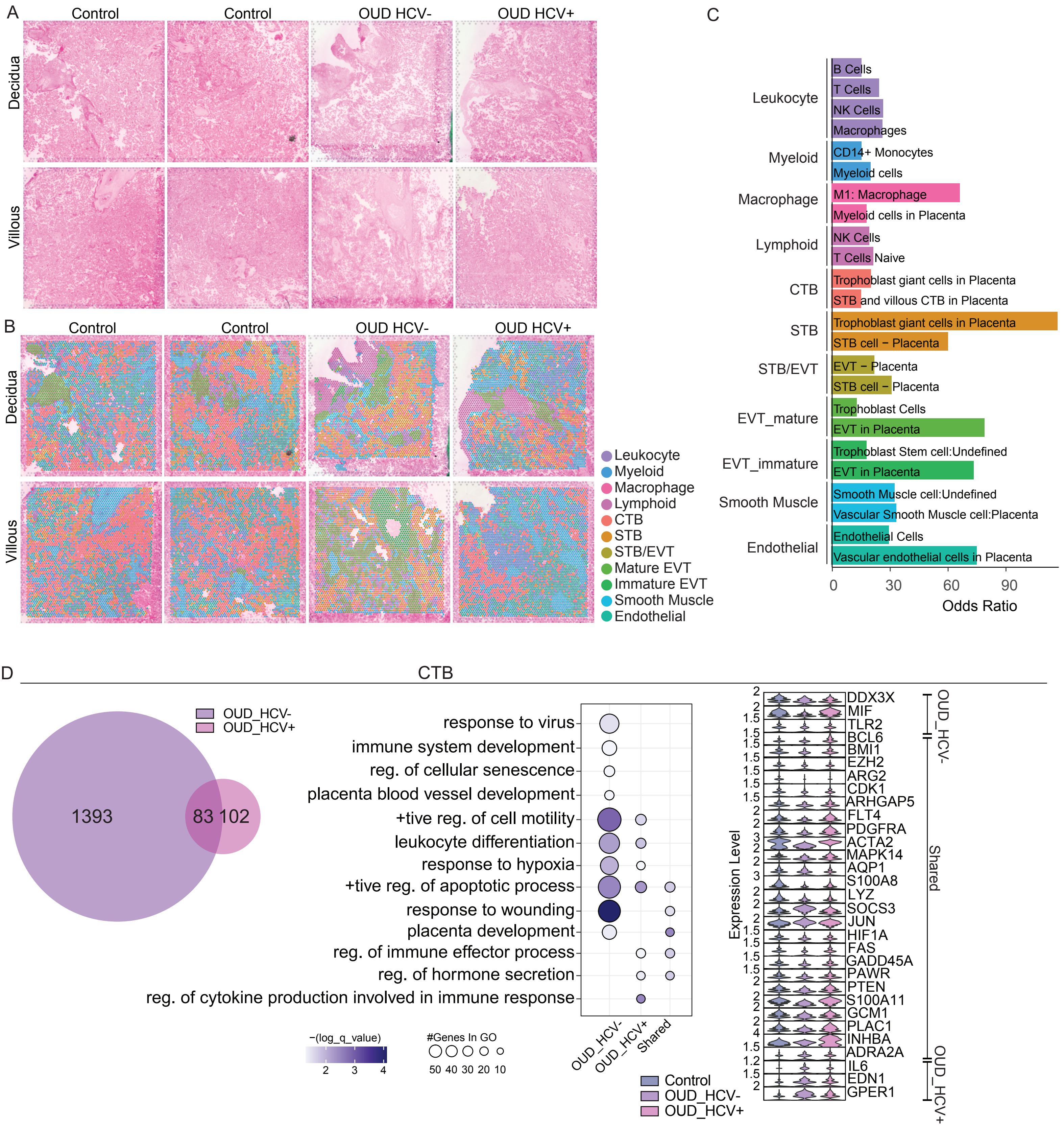

Supplemental Figure 6

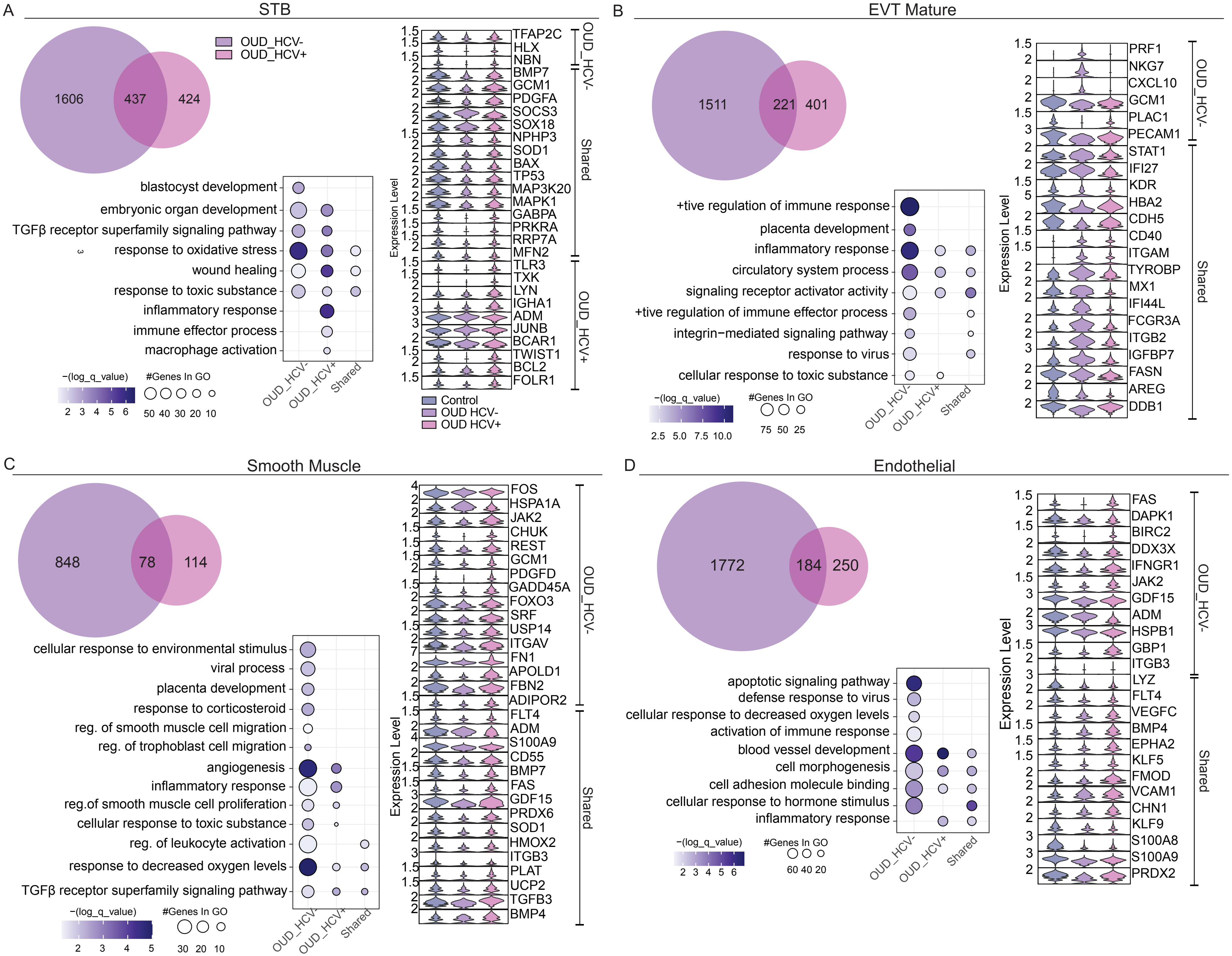

Supplemental Figure 7

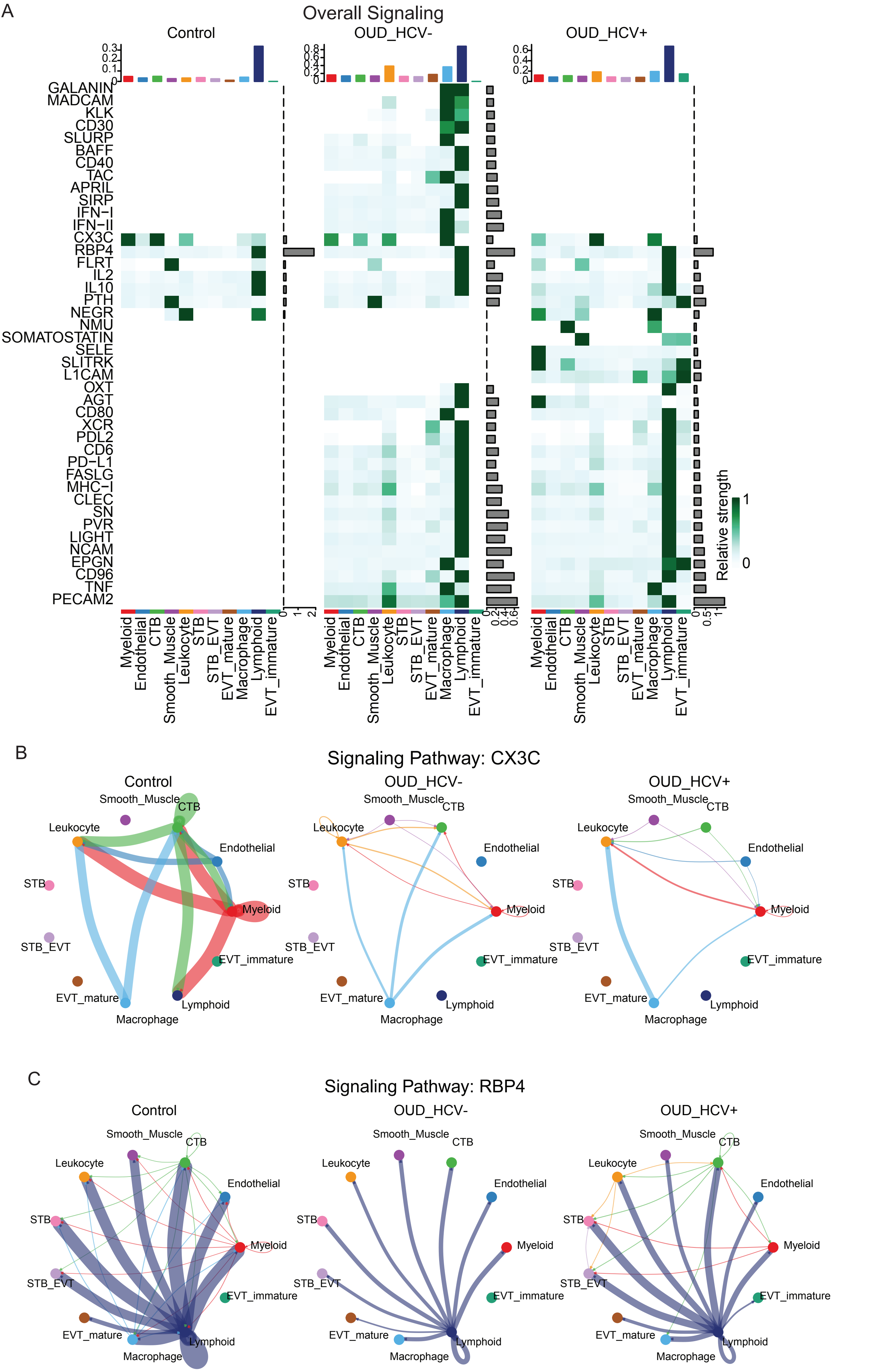

Supplement: Supplemental data [file jciinsight-11-199606-s229.pdf]
